# Antimony–Oxo Porphyrins as Photocatalysts for Redox-Neutral C–H to C–C Bond Conversion

**DOI:** 10.1021/acscatal.0c02250

**Published:** 2020-07-20

**Authors:** Luca Capaldo, Martin Ertl, Maurizio Fagnoni, Günther Knör, Davide Ravelli

**Affiliations:** † PhotoGreen Lab, Department of Chemistry, University of Pavia, Viale Taramelli 12, 27100 Pavia, Italy; ‡ Institute of Inorganic Chemistry, Johannes Kepler University Linz (JKU), Altenberger Strasse 69, 4040 Linz, Austria

**Keywords:** C−C bond formation, hydrogen atom transfer, photocatalysis, porphyrins, radical reactions

## Abstract

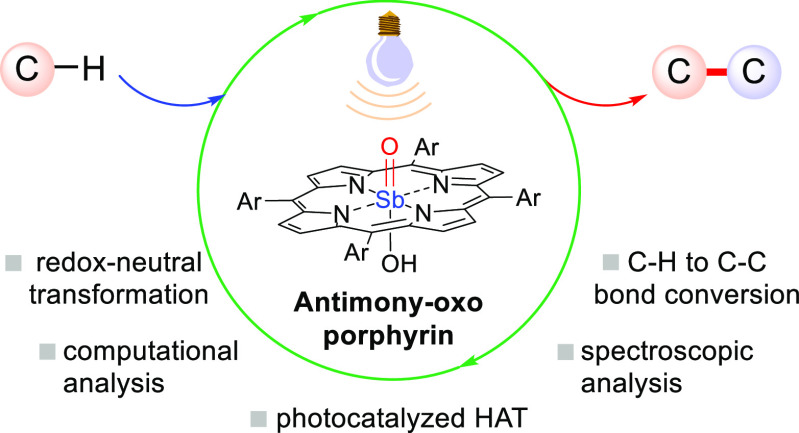

The use of high-valent antimony–oxo porphyrins as visible-light photocatalysts operating via direct hydrogen atom transfer has been demonstrated. Computational analysis indicates that the triplet excited state of these complexes shows an oxyl radical behavior, while the Sb^V^ center remains in a high-valent oxidation state, serving uniquely to carry the oxo moiety and activate the coordinated ligands. This porphyrin-based system has been exploited upon irradiation to catalyze C–H to C–C bond conversion via the addition of hydrogen donors (ethers and aldehydes) onto Michael acceptors in a redox-neutral fashion without the need of any external oxidant. Laser flash photolysis experiments confirmed that the triplet excited state of the photocatalyst triggers the desired C–H cleavage.

## Introduction

The development of eco-sustainable synthetic methodologies has recently received a fundamental contribution by visible-light photocatalysis.^1^ A photocatalyst (PC) can indeed activate the chosen organic substrate via single electron transfer (SET),^1,2^ hydrogen atom transfer (HAT),^3^ or energy transfer (EnT).^4^ These different modes of action typically require dedicated PCs (e.g. PC_SET_, PC_HAT_, or PC_EnT_, respectively). A single PC, however, may promote multiple pathways, as in the case of Eosin Y (a xanthene dye),^5^ known PC_EnT_ for singlet oxygen (^1^O_2_) generation, which has been recently adopted as PC_SET_^6,7^ and PC_HAT_.^8^

As part of our ongoing research activity, we are interested in exploring the inherent advantages of photocatalyzed HAT in redox-neutral C–C bond forming reactions.^3^ However, finding new candidates to trigger this chemistry under visible light irradiation is a tough task, and only a handful of colored PCs_HAT_ have been reported so far, notably Eosin Y,^8^ 5,7,12,14-pentacenetetrone,^9^ and the uranyl cation.^10^ Inspired by the chameleonic behavior of Eosin Y, we reasoned that other classes of catalysts specifically designed for SET or EnT might work as visible light absorbing PC_HAT_ as well. In particular, we decided to explore the class of porphyrin derivatives, widely employed as dyes for artificial photosynthesis^11^ and in many other fields.^12,13^ Porphyrins are commonly adopted as PC_EnT_ for ^1^O_2_ generation^14^ in photodynamic therapy,^15^ albeit applications in organic synthesis are now also recognized.^16,17^ Thus, while free-base porphyrins act (in analogy with other PC_SET_) either through a reductive^18^ or an oxidative^19^ quenching mechanism, when the porphyrin ring is coordinated to a suitable metal center, the thus-formed metalloporphyrin complex may show a different reactivity, thanks to the axial ligand sphere.

**Figure 1:**
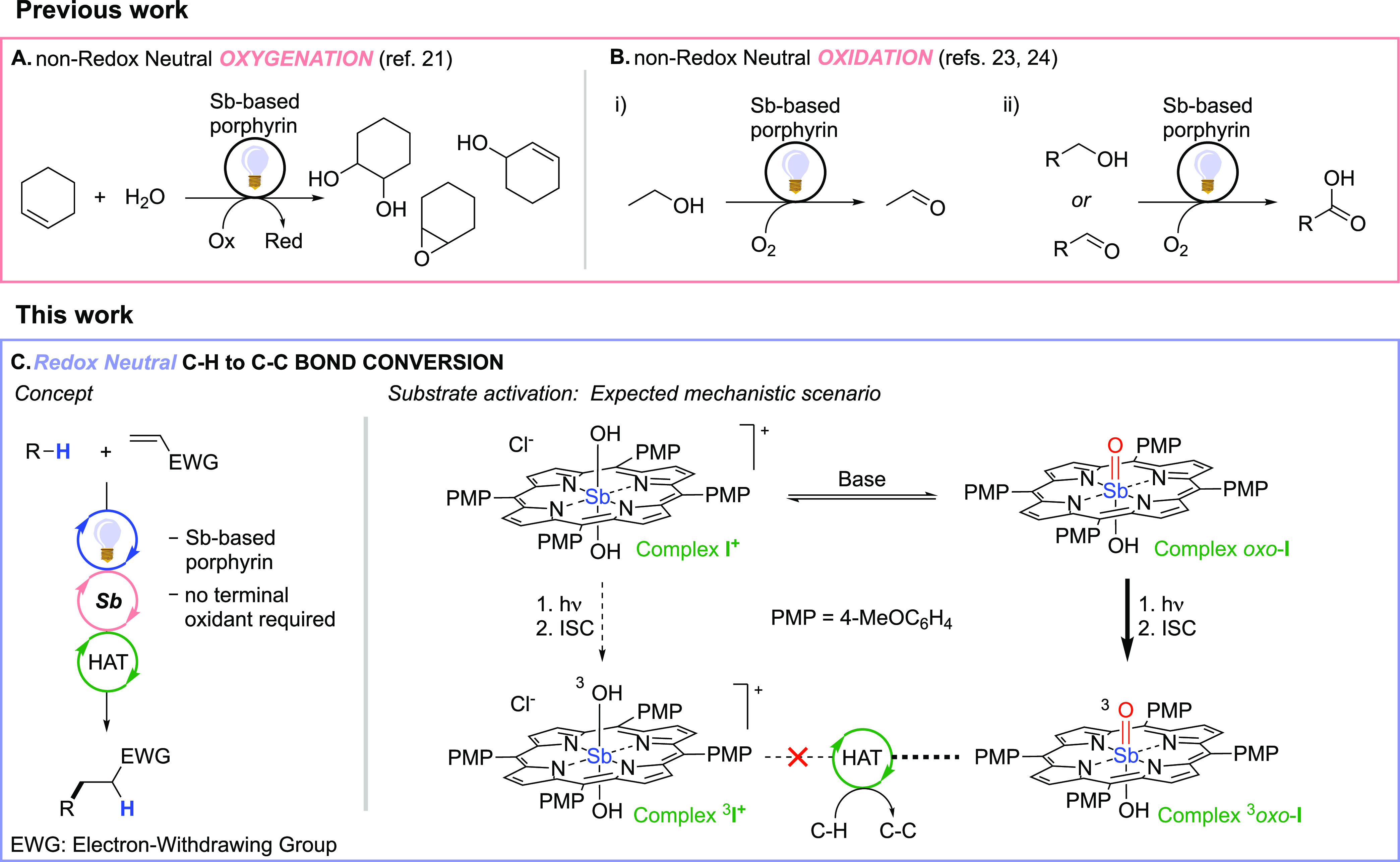
Previous examples of the use of antimony-based porphyrins as photocatalysts in non-redox neutral oxygenations (part a) and oxidations (part b), along with the new concept proposed in this work for the redox neutral C–H to C–C bond conversion and the expected mechanistic scenario (part c).

Given their low toxicity and powerful oxidation ability under photoexcitation, we selected a class of antimony dihydroxo complexes [Sb(tap)(OH)_2_]^+^X^–^ (tap = tetraarylporphyrin; X typically a halide ion or the hydroxide anion) for our purpose.^20^ These derivatives have been previously exploited to promote the photooxygenation of alkenes^21^ by using water as both the oxygen and electron donor in the presence of oxidative quenchers (e.g. methylviologen^21a−21d^ or potassium hexachloroplatinate(IV);^21e,21f^Figure 1a). Intriguingly, they are known to give an antimony–oxo complex [Sb(tap)(=O)(OH)] upon deprotonation with a base (p*K*_a_ = 9.7 for the tetraphenylporphyrin derivative).^22^ Accordingly, because currently known PCs_HAT_ are characterized by the presence of a X=O moiety (X: carbon or metal) in their structures, we reasoned that these stable porphyrin complexes may trigger the desired HAT reactivity^3^ upon light exposure. In fact, antimony–oxo porphyrins have been reported to induce the conversion of ethanol to acetaldehyde (Figure 1b, HAT proposed as the rate determining step) when exposed to visible light (546 nm).^23^ An oxyl-radical reactivity of the triplet excited state was later postulated in the aerobic photocatalyzed conversion of alcohols/aldehydes to carboxylic acids under solar light irradiation (Figure 1b).^24^

For the present work, we started looking at antimony-based porphyrins from a new perspective. While previous reports described nonredox neutral approaches for oxygenations/oxidations requiring the use of external oxidants,^21,23,24^ we hereby document the competency of this class of photocatalysts in the redox neutral C–H to C–C bond conversion under oxidant-free conditions. We also explain which species is competent in the HAT step and the features of the photocatalyst in the deactivated form, generated upon cleavage of the relevant C–H bond. Because of its intense and broad absorption bands in the visible region,^25,26^ we focused our attention on the tetra(*p*-methoxyphenyl)-substituted metalloporphyrin **I**^**+**^ (chloride salt, PMP = 4-MeOC_6_H_4_; Figure 1c).

## Results and Discussion

### Computational Analysis

At the onset of our project, we undertook a computational analysis intended to describe the different forms of the employed photocatalyst possibly involved in the process and their competency, when appropriate, in triggering the desired HAT step. Thus, previous computational investigations on the reactivity of high-valent Fe- and Mn-based porphyrins highlighted that hydrocarbon activation may occur via hydrogen abstraction by either the metal-hydroxo or the metal–oxo group.^27^ Accordingly, we initially considered both the cationic complex **I**^**+**^ and the corresponding oxo-derivative obtained via deprotonation (tagged as *oxo*-**I**; no charge present; Figure 1c). We adopted a theoretical approach based on density functional theory (DFT) simulations at the ωB97XD/def2SVP level of theory (see Supporting Information for details), well-known to correctly handle the modeling of metal complexes.^28^

We investigated the structures of **I**^**+**^ and *oxo*-**I** in the gas phase, and the corresponding optimized structures are reported in the Supporting Information (see Figure S1), clearly showing the presence of a significantly shorter Sb–O bond in *oxo*-**I** (Sb=O: 1.84 Å vs Sb–OH: 2.00 Å), indicative of a double bond character. Next, we performed a time-dependent (TD) DFT study to simulate the UV–vis spectrum of both **I**^**+**^ and *oxo*-**I**, with the final aim to collect information about the relevant electronic transitions connected with excitation of the chromophore and the involved orbitals. Notably, the TD-DFT simulations performed in acetonitrile on the previously optimized structures of **I**^**+**^ and *oxo*-**I** indicate that these two forms show very similar spectra, and a close agreement between the simulated and the experimental spectrum of *oxo*-**I** was found, further confirming the accuracy of the computational method used (Figure S2). Careful inspection of the orbitals implicated in these transitions reveals that they mainly involve the displacement of electronic density within the aromatic π-system of the tetrapyrrole ring without a significant contribution of the Sb-center and its ligands. These results are consistent with the experimental data typically obtained for high-valent antimony porphyrins, which can be classified as normal type metalloporphyrin complexes with regular electronic absorption spectra dominated by intraligand π–π* transitions of the porphyrin macrocycle.^29^

**Figure 2:**
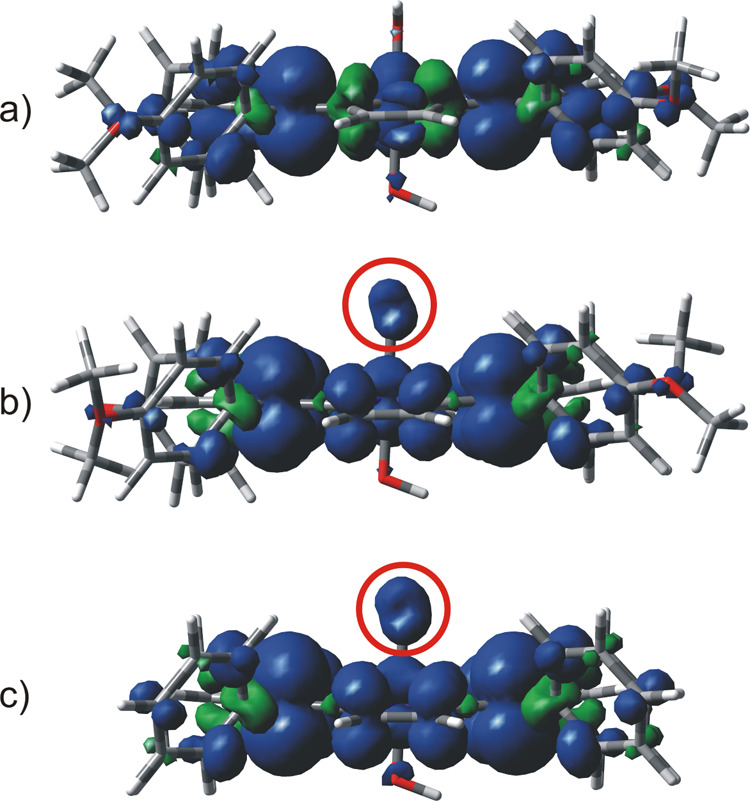
Spin density plots of the lowest lying triplet state of (a) ^3^**I**^+^, (b) ^3^*oxo*-**I**, and (c) ^3^*oxo*-**Ia** at the UωB97XD/def2SVP level of theory in the gas phase (side view).

Because it is well-known that HAT reactivity is typical of triplet excited states^30^ and previous studies demonstrated that the photoreactivity of Sb-based porphyrins involves this spin manifold,^21,23,24^ we also optimized the structures of the lowest lying triplet state of ^3^**I**^**+**^ and ^3^*oxo*-**I** because they could be populated upon intersystem crossing (ISC) from the first formed singlet excited state(s). At variance with the above, ^3^**I**^**+**^ and ^3^*oxo*-**I** show markedly different features in terms of the electronic structure. In ^3^*oxo*-**I**, inspection of the two singly occupied molecular orbitals (SOMOs) reveals that one electron is located on the π-system of the porphyrin ring, while the second one is centered on the oxygen atom of the oxo group, as we postulated. On the other hand, both SOMOs in ^3^**I**^**+**^ show electronic density only at the porphyrin ring and the *p*-methoxyphenyl substituents (see Figure S3). This difference is further corroborated by the spin density plots reported in Figure 2, where a contribution by the oxo ligand is apparent only for ^3^*oxo*-**I**, highlighting the presence of an unpaired electron on this site (red circle in Figure 2b). Quite interestingly, no spin localization is present at the antimony core in the analyzed triplets, with the central Sb atom remaining in the fully oxidized state. For the sake of comparison, we extended the latter analysis to the case of the parent tetraphenylporphyrin complex [Sb(tpp)(OH)_2_]^+^ (**Ia**^+^; tpp = tetraphenylporphyrin). Thus, the lowest lying triplet state ^3^*oxo*-**Ia** showed a strictly related electronic configuration to ^3^*oxo*-**I**, sharing the same features observed above in terms of the SOMO location (Figure S3) and spin density plot (Figure 2c).

**Figure 3:**
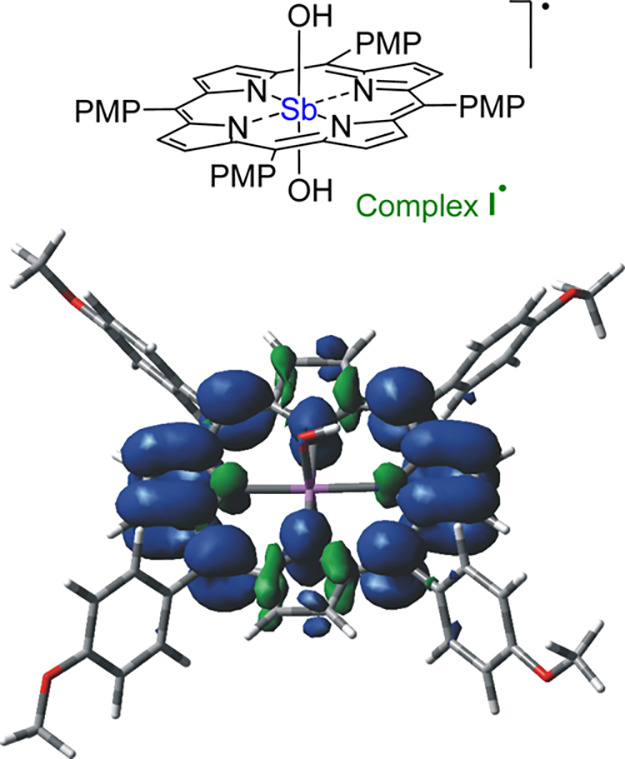
Spin density plot for complex ^**2**^**I**^**•**^ as from the calculations at the UωB97XD/def2SVP level of theory in the gas phase. PMP = *p*-methoxyphenyl.

Next, we computationally studied the deactivated form of the photocatalyst, namely complex **I**^•^ (Figure 3), formed from ^3^*oxo*-**I** through a HAT step. It is worth mentioning here that this species is a neutral doublet (^2^I^•^); its optimized structure can be found in Figure S1, while the corresponding spin density plot is reported in Figure 3. Also in this case, no spin localization can be observed at the Sb-center, which remains in the Sb^V^ oxidation state and the π-system of the porphyrin ring is the only responsible acceptor site for the extra electron present in complex **I**^•^. In other words, the redox isomeric π-radical anion form of the reduced antimony porphyrin complex and not a Sb^IV^ radical intermediate is involved in the process,^22^ which is consistent with a ligand-to-ligand charge transfer character of the reactive triplet excited state of ^3^*oxo*-**I**, as also reflected by the spin-density plot of the photoactivated complex (see Figure 2b above).

### Experimental Data on the Use of Antimony–Oxo Porphyrins as PC_HAT_

In view of these encouraging computational results, we decided to test the potential of antimony porphyrins as visible-light PC_HAT_ to promote the redox-neutral C–H to C–C conversion via a homolytic cleavage in organic substrates (Section 2.1 in the Supporting Information). As depicted in Table 1, we initially studied the addition of tetrahydrofuran (THF; **1a**) onto dimethyl maleate (**2a**) in the presence of complex **I**^**+**^, under simulated sunlight irradiation (SolarBox equipped with a 1.5 kW Xe Lamp, light intensity 500 W·m^–2^). When an acetonitrile solution of **2a** (0.05 M), **1a** (0.5 M, 10 equiv) and complex **I**^**+**^ (1 mol %) in a 1 mm optical path cuvette was irradiated for 48 h, no consumption of olefin was observed, and the desired succinate **3** was not detected (entry 1). However, shifting to MeCN/H_2_O 95:5 along with the addition of 1 mol % NaOH led to the formation of **3** (60% gas chromatography (GC) yield, based on 67% consumption of **2a**, entry 2). Varying the catalyst loading (in the 0.2–2 mol % range) confirmed that 1 mol % was the optimal choice (entries 3–5). Increasing the amount of water had a detrimental effect (entry 6), while the presence of oxygen completely inhibited the process (entry 7). Blank experiments demonstrated that both light and the catalyst are necessary (entries 8–9). To further promote the reactivity, we decided to use the more electrophilic dimethyl fumarate (**2b**)^31^ as the radical trap, which allowed the preparation of **3** in 77% yield after 24 h irradiation (entry 10). On the other hand, if complex **I**^+^ was replaced by the analogous tetraphenylporphyrin complex **Ia**^**+**^, a slightly diminished yield of **3** (66%) was observed (entry 11). In analogy to what observed in entry 1 for **I**^**+**^, no product **3** was detected when using complex **Ia**^**+**^ if base was omitted (entry 12). The result from entry 10 gave the opportunity to evaluate the effect of the light source by choosing different wavelengths, according to the absorption bands in the UV–vis spectrum of *oxo*-**I** (Figure S2).

**Table 1:**
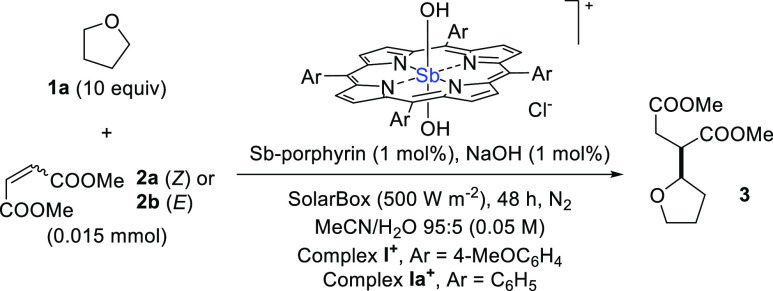
Investigation on the Photocatalyzed Addition of THF (**1a**) Onto Electron-Poor Olefins (**2**) in the Presence of Antimony-Based Porphyrin Complexes^a^

entry	olefin	Sb-based porphyrin	variation from optimized conditions	consumption (%)	yield (%)^b^
1	**2a**	**I**^**+**^	no base (NaOH)	0	n.d.
2	**2a**	**I**^**+**^	none	67	60
3	**2a**	**I**^**+**^	catalyst, base loading: 0.2 mol %	19	11
4	**2a**	**I**^**+**^	catalyst, base loading: 0.4 mol %	18	15
5	**2a**	**I**^**+**^	catalyst, base loading: 2.0 mol %	32	28
6	**2a**	**I**^**+**^	MeCN/H_2_O 1:1	90	43
7	**2a**	**I**^**+**^	air-equilibrated conditions	15	traces
8	**2a**	**I**^**+**^	in the dark	0	n.d.
9	**2a**	**I**^**+**^	no photocatalyst	0	n.d.
10	**2b**	**I**^**+**^	24 h irradiation	100	77
11	**2b**	**Ia**^**+**^	24 h irradiation	100	66
12	**2b**	**Ia**^**+**^	no base (NaOH)	0	n.d.
13	**2b**	**I**^**+**^	irradiation wavelength: 366 nm	0	n.d.
14	**2b**	**I**^**+**^	irradiation wavelength: 405 nm	80	70
15	**2b**	**I**^**+**^	irradiation wavelength: 455 nm	53	70
16	**2b**	**I**^**+**^	irradiation wavelength: 589 nm	0	n.d.

a Conditions: reaction performed in a 1 mm cuvette on a 300 μL nitrogen-purged solution containing **1a** (10 equiv), **2** (0.05 M) and complex **I**^**+**^ or **Ia**^**+**^ (1 × 10^–4^ to 1 × 10^–3^ M, 0.2–2.0 mol %); the corresponding *oxo*-**I** or *oxo*-**Ia** complexes are generated *in situ* in the presence of NaOH in the chosen reaction medium (see Supporting Information, method B).

b GC yields referred to the consumption of the limiting reagent (**2**), using *n*-dodecane as the internal standard; n.d.: not detected.

Thus, irradiation of complex *oxo*-**I** with ten 15 W phosphor-coated lamps (λ_EM_ centered at 366 nm) under the optimized conditions did not lead to any appreciable consumption of **2b** (entry 13). The situation changed dramatically, however, when employing monochromatic visible-light LEDs (1 W) with emission centered at 405 or 455 nm because we consistently observed the formation of product **3** in 70% yield, albeit in the former case a higher conversion of **2b** (80 vs 53%) was found (compare entries 14 and 15). Furthermore, we also tested a sodium vapour lamp (emission centered at 589 nm), but the olefin remained untouched (entry 16).

Next, we tested the catalytic system on a bigger scale (0.05 mmol), making use of more powerful visible light irradiation sources (405 or 455 nm LEDs, 18 W) to extend the optimized protocol to different reaction partners (Table 2). Gratifyingly, we found excellent yields in most of the tested cases and, in general terms, the reactions proceeded faster under 405 nm than 455 nm irradiation. Thus, the addition of **1a** onto **2b** afforded product **3** in 91 and 44% yield, respectively, upon irradiation for 48 h, albeit only a partial conversion of the starting materials was observed. Similarly, **1a** could be smoothly added onto 2-cyclohexylidenemalononitrile (**2c**) and 2-benzylidenemalononitrile (**2d**) to give **4** and **5**, respectively, in yields up to 82%. Finally, the feasibility of a C(sp^2^)–H cleavage was verified by adopting heptaldehyde **1b** as the substrate. Also in this case, compounds **6** and **7** were obtained in good yields from the reaction with **2b** and **2d**, respectively. On the other hand, no product formation was observed when the challenging alkylation of **2b** with cyclohexane **1c** as the H-donor was attempted (data not shown).

**Table 2:**
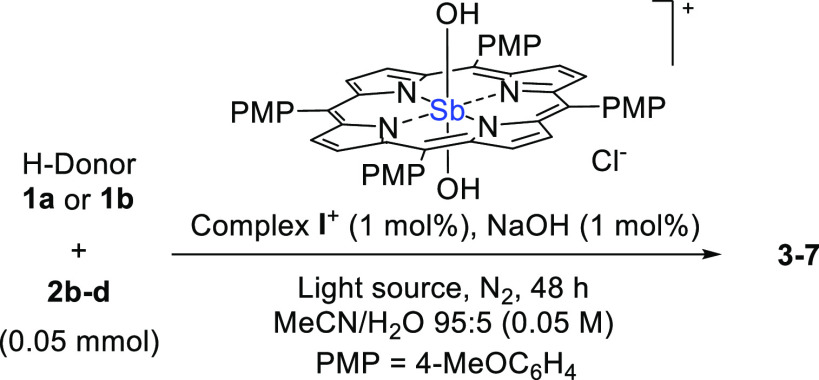

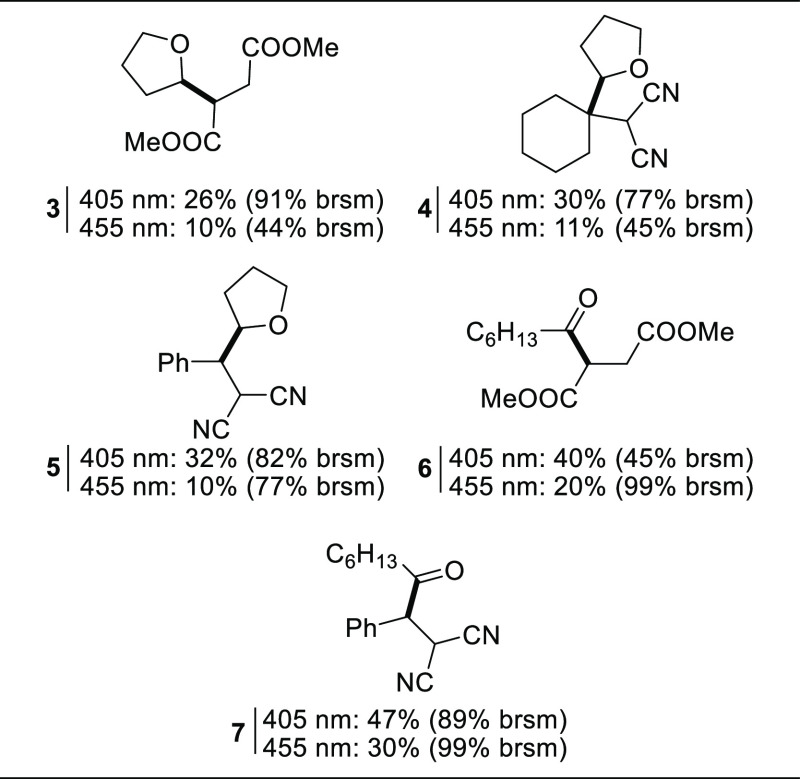
Investigation on the Photocatalyzed Addition of Different Hydrogen Donors (**1a,b**) Onto Electron-Poor Olefins **2b-d** in the Presence of Antimony–Porphyrin Complex **I**^**+**^^a^^,^^b^

a **1a**: tetrahydrofuran; **1b**: heptaldehyde; **2b**: dimethyl fumarate; **2c**: 2-cyclohexylidenemalononitrile; and **2d**: 2-benzylidenemalononitrile.

b Conditions: reaction performed in a 1 dram vial on a 1 mL nitrogen-purged solution containing **1** (10 equiv for **1a**; 1 equiv for **1b**), **2** (0.05 M) and *oxo*-**I** (5 × 10^–4^ M, 1.0 mol %) in MeCN/H_2_O 95:5 (see Supporting Information, method A). GC yields referred to the consumption of the limiting reagent (**2**), using *n*-dodecane as the internal standard. Brsm: based on remaining starting materials.

### Mechanistic Investigation

**Scheme 1:**
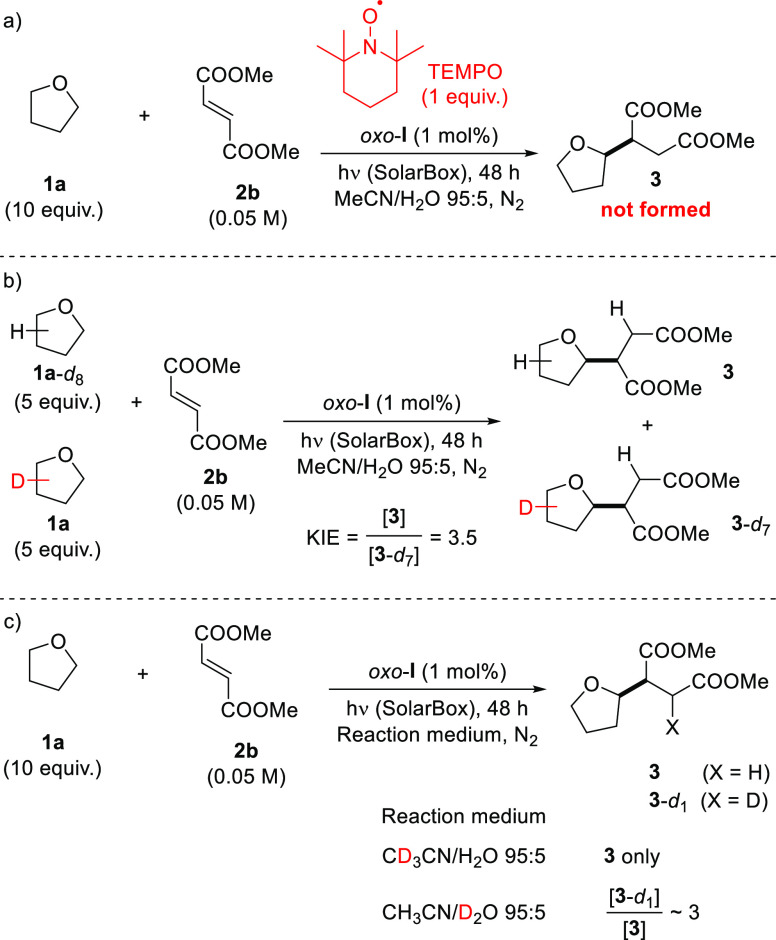
Chemical Quenching Experiments (Part a) and Isotopic Labeling Studies in the Reaction between **1a** and **2b**: Cross-Over Experiment (Part b); Use of the Deuterated Media (Part c)

The radical nature of the process can be inferred from its inhibition in the presence of TEMPO ((2,2,6,6-tetramethylpiperidin-1-yl)oxyl; Scheme 1a). Building on this result, we then performed isotopic labelling experiments to have insights into the mechanistic scenario and the results are gathered in Scheme 1 (see also Sections 2.2 and 2.3 in the Supporting Information). When the reaction between THF and **2b** was performed by adopting an equimolar mixture of **1a** and perdeuterated **1a**-*d*_8_ (5 equiv each), a preferential activation of the former occurred, with formation of **3** and **3**-*d*_7_ in a 3.5:1 ratio (Scheme 1b) according to GC–MS analysis (see Supporting Information for details). Next, we repeated the experiment in the presence of a deuterated medium, to verify if the solvent had a role. When the experiment was run in a CD_3_CN/H_2_O mixture, no deuterium was incorporated in the obtained product, while a significant deuteration of the 3-position of **3** was observed when adopting CH_3_CN/D_2_O as the medium (Scheme 1c).

Another point worth to be investigated relates to the activation mechanism of H-donors by the excited porphyrin complex. Previous studies indicated that the triplet excited state of Sb-based tetraarylporphyrins is able to oxidize monoelectronically the hydroxide anion to the hydroxyl radical HO^•^,^21c^ a potent oxidant able to trigger an indirect photocatalytic HAT process.^3b^ However, this pathway has been reported to occur in the presence of a large excess of the base (500-fold),^21c^ while an equimolar amount of NaOH with respect to antimony porphyrin is added under our conditions to form *oxo*-**I**. Nonetheless, with the final aim to safely exclude a role for this indirect pathway and to further elucidate the nature of the light-mediated primary steps, we carried out excited state quenching experiments with THF (**1a**) as the H-donor (Section 2.4 in the Supporting Information). Upon addition of increasing amounts of **1a** to samples of the active photocatalyst *oxo*-**I**, no indication of the singlet excited state quenching was observed, as monitored by fluorescence quantum yield measurements (data not shown). On the other hand, a nanosecond laser flash photolysis study of the triplet excited state manifold clearly confirmed that a dynamic bimolecular quenching process between the photocatalyst and **1a** was taking place.

**Figure 4:**
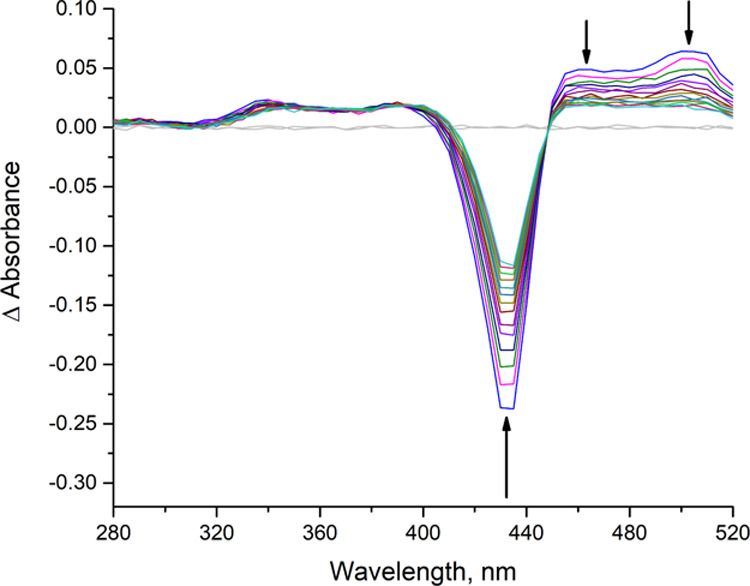
Transient differential absorption spectra obtained upon nanosecond flash photolysis (532 nm) of *oxo*-**I** in degassed CH_3_CN/H_2_O 95:5 showing the spectral variations occurring within the first 17 μs after the laser flash. The flat lines in gray represent the pre-pulse signals.

Time-resolved spectra of the photoexcited catalyst *oxo*-**I** in the absence of a quencher are shown in Figure 4 (see also Figure S9 for a longer timescale). Bleaching of the ground state absorption spectrum giving rise to a negative Soret-band signal between 400 and 440 nm is accompanied by an arising transient signal with a peak around 460 nm, which is characteristic for the triplet excited state of regular tetraarylporphyrin metal complexes.^32,33^ In addition, the triplet–triplet absorption spectrum of the antimony–oxo complex in the given solvent mixture displays a second conspicuous signal with a maximum at 510 nm (Figure 4), which decays with a lifetime of (8.0 ± 0.1) μs, in agreement with a significant charge-transfer character of the triplet excited state manifold of ^3^*oxo*-**I**.

**Figure 5:**
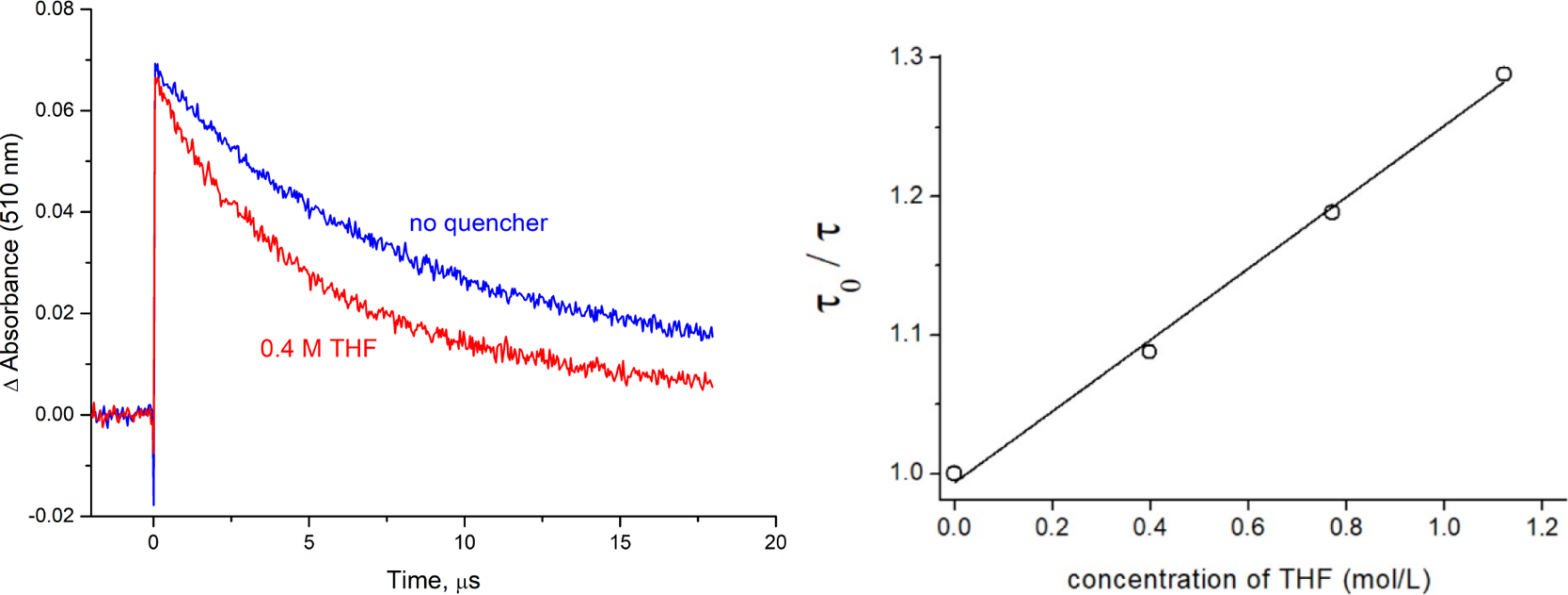
Left side: time absorption profiles recorded at 510 nm, monitoring the monoexponential decay process of the lowest excited triplet state of *oxo*-**I** in degassed CH_3_CN/H_2_O 95:5 in the absence (blue line) and presence (red line) of THF. Right side: Photokinetic data analysis indicates that bimolecular quenching of the *oxo*-**I** triplet state manifold by the hydrogen atom donor THF occurs.

The deactivation rate of the lowest triplet excited state of *oxo*-**I** significantly increases upon addition of **1a**, clearly acting as a quencher (Figure 5). The corresponding Stern–Volmer plot is linear in the tested concentration range (up to 1 M THF) and yields a slope of *K*_SV_ = (0.26 ± 0.01) M^–1^ at 298 K in agreement with a dynamic bimolecular process. From the corresponding lifetime data, a quenching rate constant of *k*_Q_ = (3.3 ± 0.2) × 10^4^ M^–1^·s^–1^ was obtained.

**Scheme 2:**
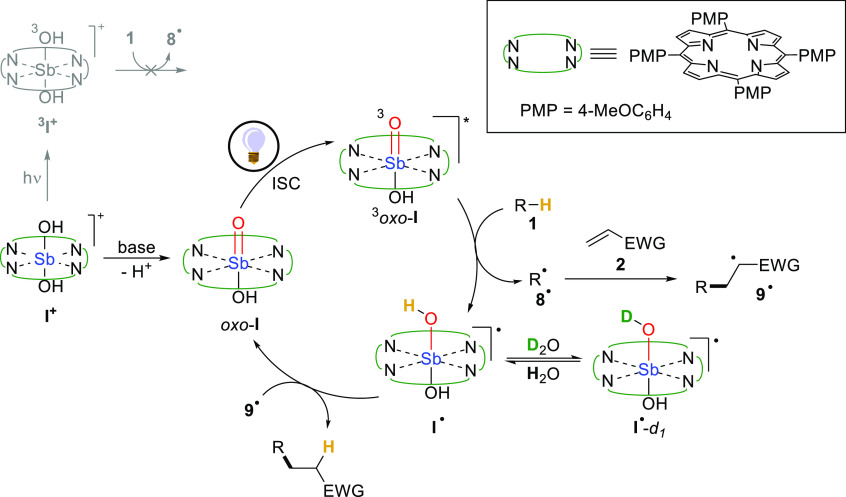
Proposed Mechanism. EWG: Electron-Withdrawing Group

Based on the above reported computational and experimental evidence, we propose that the present photocatalytic process occurs as depicted in Scheme 2 for the case of the tetra(*p*-methoxyphenyl)-substituted metalloporphyrin. Thus, dihydroxo complex **I**^**+**^ is not active as PC_HAT_, however, upon addition of a stoichiometric amount of base (aqueous NaOH), the antimony–oxo functionality of *oxo*-**I** is generated *in situ*. Following visible light absorption and ISC, the triplet excited state (^3^*oxo*-**I**) endowed with a partial oxyl radical character is populated, which confirms previous speculations present in the literature.^24^ Notably, the same behavior has been confirmed for the parent ^3^*oxo*-**Ia** derivative as well. According to excited state quenching experiments, ^3^*oxo*-**I** is prone to abstract a hydrogen atom from **1** to give the C-centered radical **8**^•^, which is then trapped by **2** to afford **9**^•^. Finally, the desired Giese adduct is generated upon back-HAT or stepwise electron/proton transfer. This last step consists in the closure of the photocatalytic cycle, which also restores the active form of the photocatalyst without the need of terminal oxidants. Furthermore, according to Scheme 1c, we suggest that a ligand exchange involving a protic solvent may occur at the **I**^•^ stage to give **I**^•^-*d*_1_, explaining the partial deuteration observed when a deuterated protic medium was employed.

The hereby reported use of an oxo-porphyrin derivative to promote a redox-neutral process occurring via visible-light photocatalyzed HAT has not been previously explored. Key to the success of this chemistry is the adoption of a stable metal–oxo porphyrin complex, containing a high-valent oxidation state element of the p-block, notably antimony, for several reasons. First of all, Sb^V^ can coordinate two additional ligands (the hydroxyl groups) other than porphyrin, which is a prerequisite to install the axial oxo functionality implicated in the excited state reactivity. On top of that, high-valent antimony is superior to other elements because it exerts a peculiar electron-withdrawing electronic effect and imposes a strong polarization to the attached axial ligands and the porphyrin macrocycle, accordingly.^34^ This property is expected to have a crucial role in both controlling the pKa of the attached hydroxyl ligands and the reactivity in the triplet excited state.

The approach is remarkably different from that of iron- and manganese–oxo porphyrins known to promote hydroxylation and halogenation reactions in alkanes via a *thermal* HAT step.^35^ The latter behavior may be attributed to the presence of a partially filled *d* shell in the metal center, endowing the complex with a radicaloid character. These complexes are characterized by several low-lying, close-in-energy electronic states that mix together, giving rise to the so-called “redox mesomerism” phenomenon.^36^ Thus, these metal–oxo porphyrins trigger a thermal homolytic C–H cleavage, and the resulting C-centered radical can be used for C–X bond formation through a rebound mechanism,^35^ with the concomitant reduction of the complex (the oxidation state at M goes from *n*^+^ to (*n* – 2)^+^). Accordingly, these complexes have been conveniently exploited as catalysts in net-oxidative processes, where the presence of a terminal oxidant is mandatory for the formation of the oxo-species. This peculiar characteristic makes these complexes unsuitable for other types of chemistry, such as the redox-neutral process presented herein.

## Conclusions

Starting off from computational investigation, we experimentally demonstrated that a thermally unreactive Sb^V^–oxo porphyrin can be exploited to trigger a redox-neutral C–H to C–C bond conversion based on a photocatalytic HAT step occurring under visible light irradiation. We also proved the radical nature of the process via inhibition experiments performed in the presence of TEMPO, while the involvement of a HAT step is corroborated by the observed kinetic isotopic effect. Meticulous spectroscopic investigation allowed to identify the nature of the activation step and to determine the quenching constant value, as well.

Finally, according to our computational analysis, the Sb^V^ center remains in the high-valent oxidation state under the conditions explored, serving uniquely to carry the oxo moiety and to activate the coordinated ligands. Thus, antimony has a spectator role in the key steps of the photocatalytic cycle, suggesting that other high-valent porphyrin complexes featuring an oxo ligand may be envisaged as PC_HAT_. Experiments in this direction are ongoing in our laboratories.

## Supporting Information Available

Experimental details about the used materials; sample preparation; mechanistic experiments; and computational data (including optimized structures) (PDF)
