# Supplementary material for: Antimony–Oxo Porphyrins as Photocatalysts for Redox-Neutral C–H to C–C Bond Conversion
Source: ACS Catal. 2020 Jul 20;10(16):9057–64. doi: 10.1021/acscatal.0c02250 (PMC8009479; doi:10.1021/acscatal.0c02250)
Supplement: Supplementary file 1 — cs0c02250_si_001.pdf [file cs0c02250_si_001.pdf]

# Supporting Information

## Antimony-Oxo Porphyrins as Photocatalysts for Redox-Neutral C–H to C–C Bond Conversion

Luca Capaldo,[†] Martin Ertl,[‡] Maurizio Fagnoni,[†] Günther Knör,[‡,*] and Davide Ravelli[†,*]

[†] *PhotoGreen Lab, Department of Chemistry, University of Pavia, viale Taramelli 12, 27100 Pavia, Italy*. e-mail: davide.ravelli@unipv.it

[‡] *Institute of Inorganic Chemistry, Johannes Kepler University Linz (JKU), Altenberger Strasse 69, 4040 Linz, Austria.* e-mail: guenther.knoer@jku.at

## CONTENT


1. **Computational Details** **p. S2**
   - **1.1 Methods** **p. S2**
   - **1.2 DFT optimized structures** **p. S2**
   - **1.3 UV-Vis spectra simulation** **p. S4**
   - **1.4 Singly Occupied Molecular Orbitals (SOMO)** **p. S8**
   - **1.5 Cartesian coordinates** **p. S9**
2. **Experimental Details** **p. S19**
   - **2.1 General information** **p. S19**
   - **2.2 Chemical quenching experiments** **p. S22**
   - **2.3 Deuterium labeling experiments** **p. S23**
   - **2.4 Photochemical quenching experiments** **p. S26**
3. **References** **p. S28**

# 1) Computational Details

## 1.1 Methods

All the calculations were carried out using the Gaussian16 program package[S1] at the CINECA Supercomputer center (Italy). In our investigation, all the structures have been optimized having recourse to density functional theory (DFT), *viz.* adopting the ωB97XD functional with an unrestricted (U) formalism when systems containing an unpaired number of electrons were considered, and the standard def2SVP basis set in the gas phase. To confirm the nature of the optimized structures, vibrational frequencies have been calculated at the same level of theory as geometry optimizations, and it was verified that they had only real frequencies.

For each of the reported structures, a systematic investigation of all of the possible conformations has been carried out. However, only the most stable conformation has been reported and has been considered for further work.

The solvent effect was included by single-point calculations on the optimized geometries obtained in the gas phase at the (U)ωB97XD/def2SVP level of theory in acetonitrile bulk, by maintaining the default solvent options of the SMD model.

Time-Dependent DFT (TD-DFT) has been performed via the “TD” keyword by calculating the 15 lowest singlet transitions. The spin density plot for $^{3}$*oxo*-**I**, $^{3}$*oxo*-**Ia** and **I$^{•}$** have been determined via the “cubegen” command.

## 1.2 DFT optimized structures

Figure S1 shows the geometries of: **I$^{+}$** (part a) and *oxo*-**I** (part b) in the singlet ground state and those of the corresponding triplet excited states **$^{3}$I$^{+}$** (part c) and **$^{3}$***oxo*-**I** (part d), as well as that of the doublet species **I$^{•}$** (part e). All the structures have been optimized at the (U)ωB97XD/def2SVP level of theory in the gas phase. As indicated, the mean Sb–N bond length is markedly shorter (around 0.05 Å) in the case of **I$^{+}$** with respect to *oxo*-**I** and a similar trend is observed for the corresponding triplet states as well. Doublet derivative **I$^{•}$** strictly resembles complex **I$^{+}$**.

Furthermore, worth of notice is the arrangement of the Sb atom in those species bearing an oxo moiety (see the case of *oxo*-**I** and $^{3}$*oxo*-**I**; parts b and d), since the metal center is consistently displaced towards the (shorter) oxo group with respect to the porphyrin ring. On the other hand, a highly symmetric structure is observed for all the remaining structures.

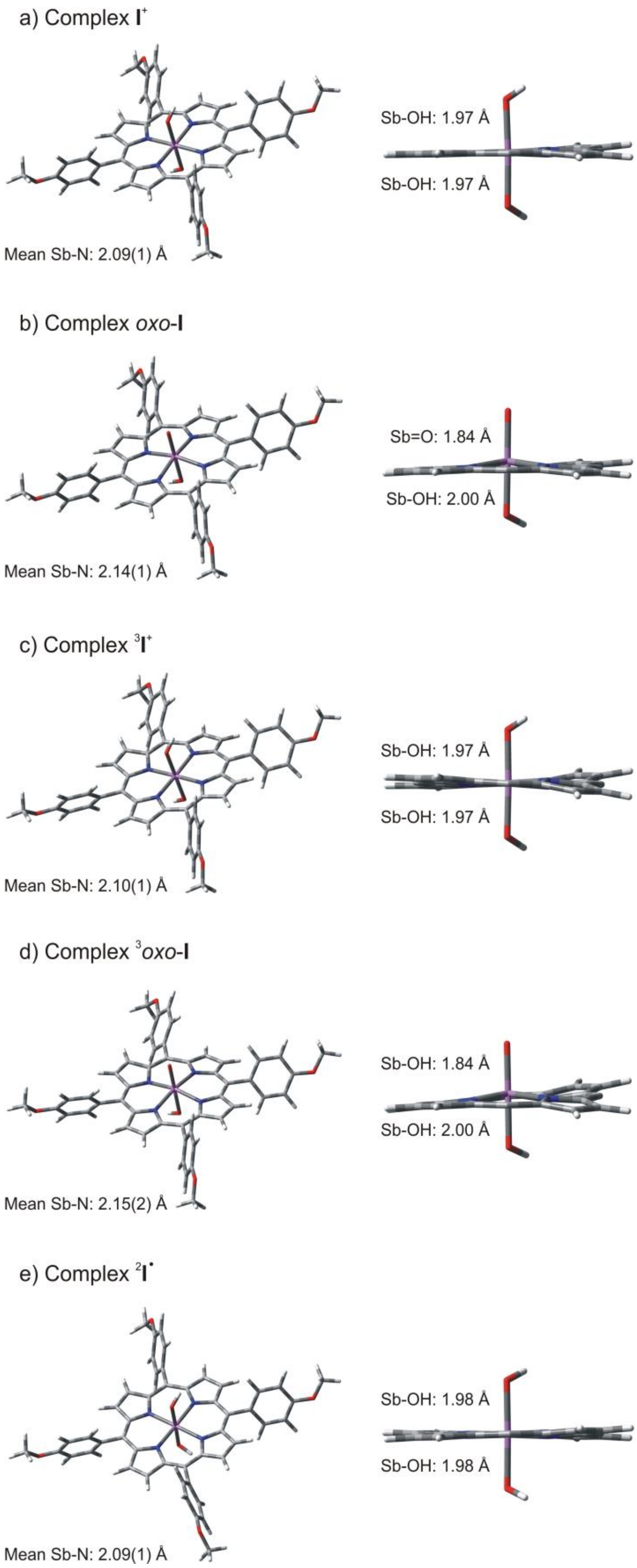


**Figure S1.** Structures of complexes **I**$^{+}$ (part a) and *oxo*-**I** (part b) in the singlet ground state and those of the corresponding triplet excited states $^{3}$**I**$^{+}$ (part c) and $^{3}$*oxo*-**I** (part d), as well as that of the doublet species **I**$^{\bullet}$ (part e) at the (U)ωB97XD/def2SVP level of theory in the gas phase. On the right: side-view of the porphyrin group only.

## 1.3 UV-Vis spectra simulation

Figure S2 gathers the registered UV-Vis spectrum of a $5\times10^{-6}$ M solution of *oxo*-**I** in MeCN/$H_2O$ 95:5, obtained by treating complex $\mathbf{I}^+$ with an equimolar amount of NaOH. As shown in the inset, the solution has a bright green color. This color results from the presence of the two small Q-bands in the 500-650 nm range and of the intense Soret band centered at 432 nm ($\varepsilon_{MAX}$ = $1.53\times10^5$ $M^{-1}\cdot cm^{-1}$).

The simulations performed on the optimized structures of singlet ground state complexes $\mathbf{I}^+$ and *oxo*-**I** according to the TD-DFT approach at the SMD-ωB97XD/def2SVP level of theory in acetonitrile are shown as well in Figure S2. These are reported in terms of vertical electronic transitions, characterized by the wavelength and the associated intensity (oscillator strength, vertical right axis). Limiting the discussion to complex *oxo*-**I**, it can be observed that there are two low-intensity transitions in the region of the Q-bands, albeit they are closer than the two maxima found in the real spectrum. On the other hand, the theory slightly over-estimates the energy of the Soret band, since very intense transitions are found at 390 nm compared to the 432 nm maximum of the registered spectrum (Δλ *ca.* 40 nm).

At any rate, by inspecting the orbitals mainly contributing to these transitions, they always seem to involve a displacement of electronic density within the π-electron system of the porphyrin core in agreement with the "four-orbital model", with no significant contribution from the orbitals of the metal center (except from orbital 212 predominately corresponding to the $a_{2u}$ (π) frontier orbital of the porphyrin ligand system with some minor admixed contribution from the central antimony-oxo moiety). Furthermore, in the four-orbital model of porphyrin spectra both $e_g$ orbitals representing the LUMOs should be degenerate, one with dominant orbital coefficients at opposite nitrogen atoms in x-axis direction and one in y-axis direction in ideal $D_{4h}$ symmetry. Since axial OH-groups are present here $D_{4h}$ symmetry is broken and ordering of the orbitals 213 and 214 slightly depends on the chosen orientation of the axial ligands.

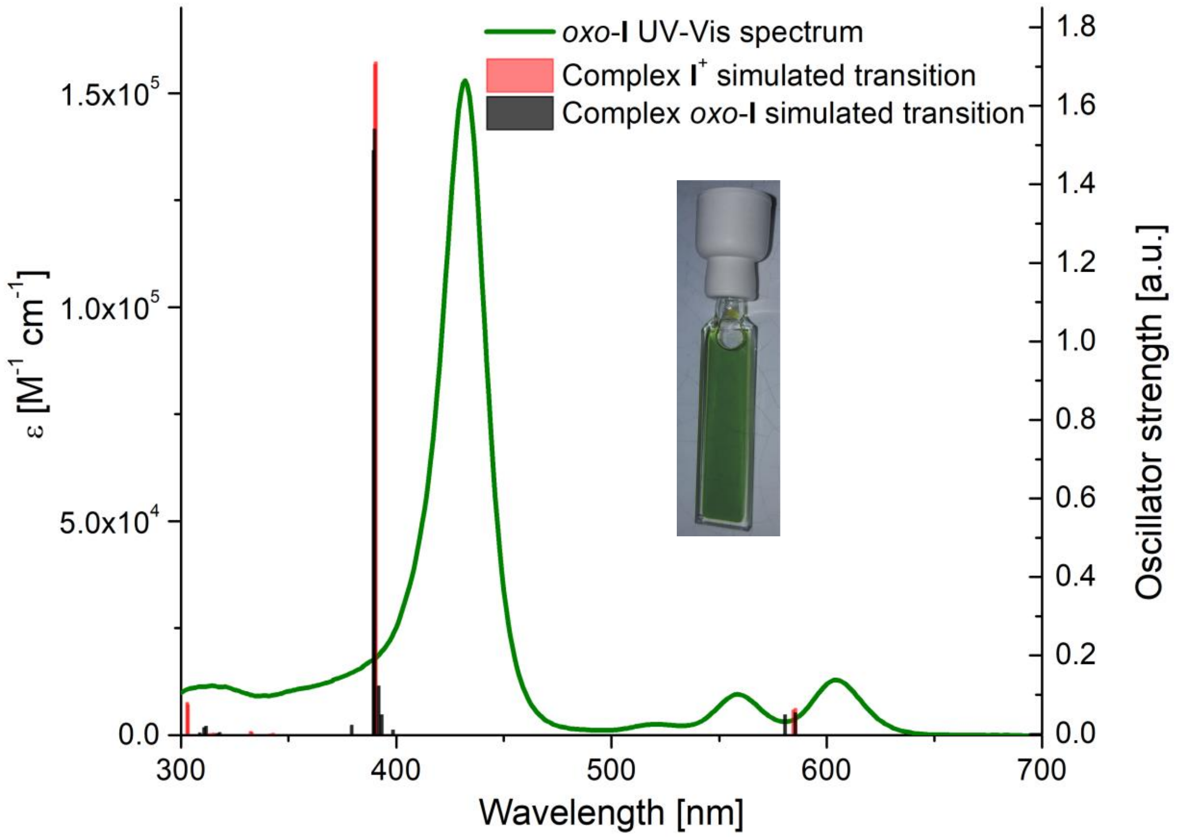


**Figure S2**. Experimental UV-Vis spectrum of complex *oxo*-**I** ($5\times10^{-6}$ M solution of in MeCN/$H_2O$ 95:5, green line, left axis); the inset shows the color of the solution. Simulated electronic transitions for ground state complexes $\mathbf{I}^+$ (red bars) and *oxo*-**I** (black bars) as from the TD-DFT calculation at the SMD-ωB97XD/def2SVP level of theory in acetonitrile (right axis; see Tables S1-S2 for details).

**Table S1**. Complex **I**$^{+}$ 15 lowest singlet transitions at the SMD-ωB97XD/def2SVP level of theory in acetonitrile.

| # | **Wavelength [nm]** | **Oscillator strength [a.u.]** | **Orbital composition** (only main contributions shown) |
|---|---|---|---|
| **1** | **585.59** | **0.0643** | 211 → 214 -0.40783<br>212 → 213 0.52839 |
| **2** | **584.65** | **0.0607** | 211 → 213 0.41016<br>212 → 214 0.52652 |
| **3** | **390.96** | **1.7110** | 211 → 214 0.55020<br>212 → 213 0.43065 |
| **4** | **390.56** | **1.7049** | 211 → 213 0.54866<br>212 → 214 -0.43272 |
| 5 | 343.24 | 0.0014 | |
| 6 | 341.95 | 0.0004 | |
| 7 | 333.36 | 0.0030 | |
| 8 | 332.87 | 0.0061 | |
| 9 | 315.57 | 0.0019 | |
| 10 | 314.05 | 0.0010 | |
| 11 | 303.74 | 0.0741 | |
| 12 | 303.5 | 0.0799 | |
| 13 | 291.59 | 0.0006 | |
| 14 | 290.27 | 0.0010 | |
| 15 | 280.98 | 0.0828 | |

Orbital **211 ($a_{1u}$)**

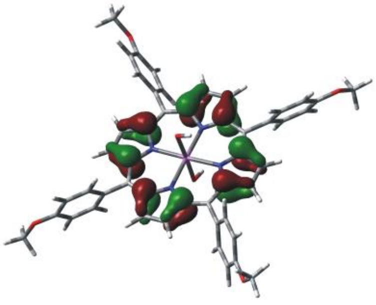

Orbital **212 ($a_{2u}$)**

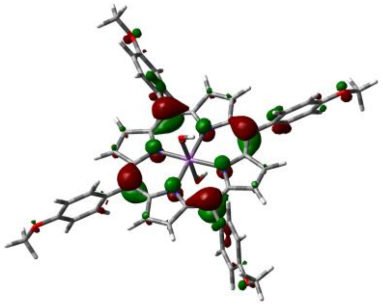

Orbital **213 ($e_{gy}$)**

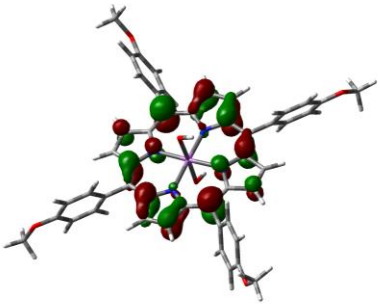

Orbital **214 ($e_{gx}$)**

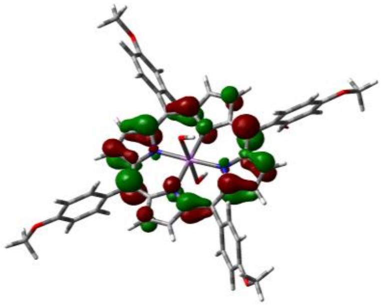

**Table S2**. Complex *oxo*-**I** 15 lowest singlet transitions at the SMD-ωB97XD/def2SVP level of theory in acetonitrile.

| # | Wavelength [nm] | Oscillator strength [a.u.] | Orbital composition (only main contributions shown) |
|---|---|---|---|
| **1** | **585.48** | **0.0534** | 211 → 214 -0.41380<br>212 → 213 0.56777 |
| **2** | **580.85** | **0.0485** | 211 → 213 0.42050<br>212 → 214 0.56272 |
| 3 | 398.92 | 0.0094 | |
| 4 | 393.74 | 0.0483 | |
| 5 | 392.28 | 0.1214 | |
| **6** | **390.30** | **1.5402** | 211 → 214 0.54174<br>212 → 213 0.39230 |
| **7** | **389.95** | **1.4856** | 211 → 213 0.52146<br>212 → 214 -0.38856 |
| 8 | 379.78 | 0.0221 | |
| 9 | 318.58 | 0.0028 | |
| 10 | 317.34 | 0.0007 | |
| 11 | 312.2 | 0.0194 | |
| 12 | 311.33 | 0.0157 | |
| 13 | 309.29 | 0.0019 | |
| 14 | 293.55 | 0.0020 | |
| 15 | 293.05 | 0.0034 | |

Orbital **211 ($a_{1u}$)**

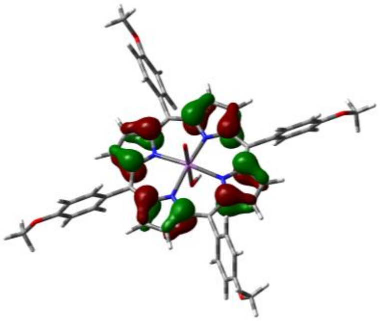

Orbital **212 ($a_{2u}$)**

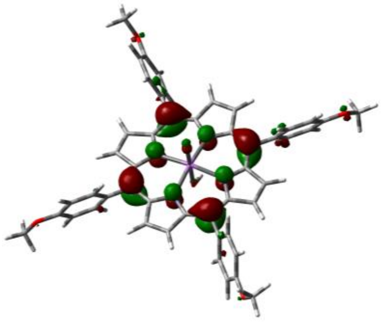

Orbital **213 ($e_{gx}$)**

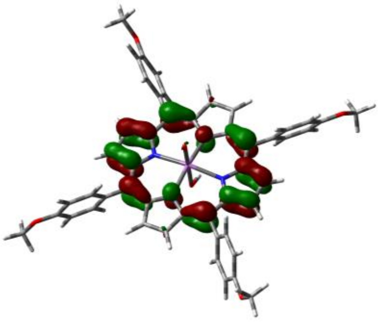

Orbital **214 ($e_{gy}$)**

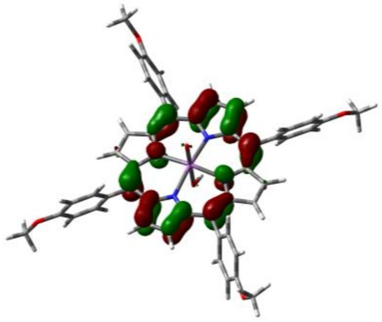

## 1.4 Singly Occupied Molecular Orbitals (SOMO)

Figure S3 gathers the Singly Occupied Molecular Orbitals (SOMO) of the lowest lying triplet state of Sb-based complexes $^{3}$**I**$^{+}$ (part a), $^{3}$*oxo*-**I** (part b) and $^{3}$*oxo*-**Ia** (part c).

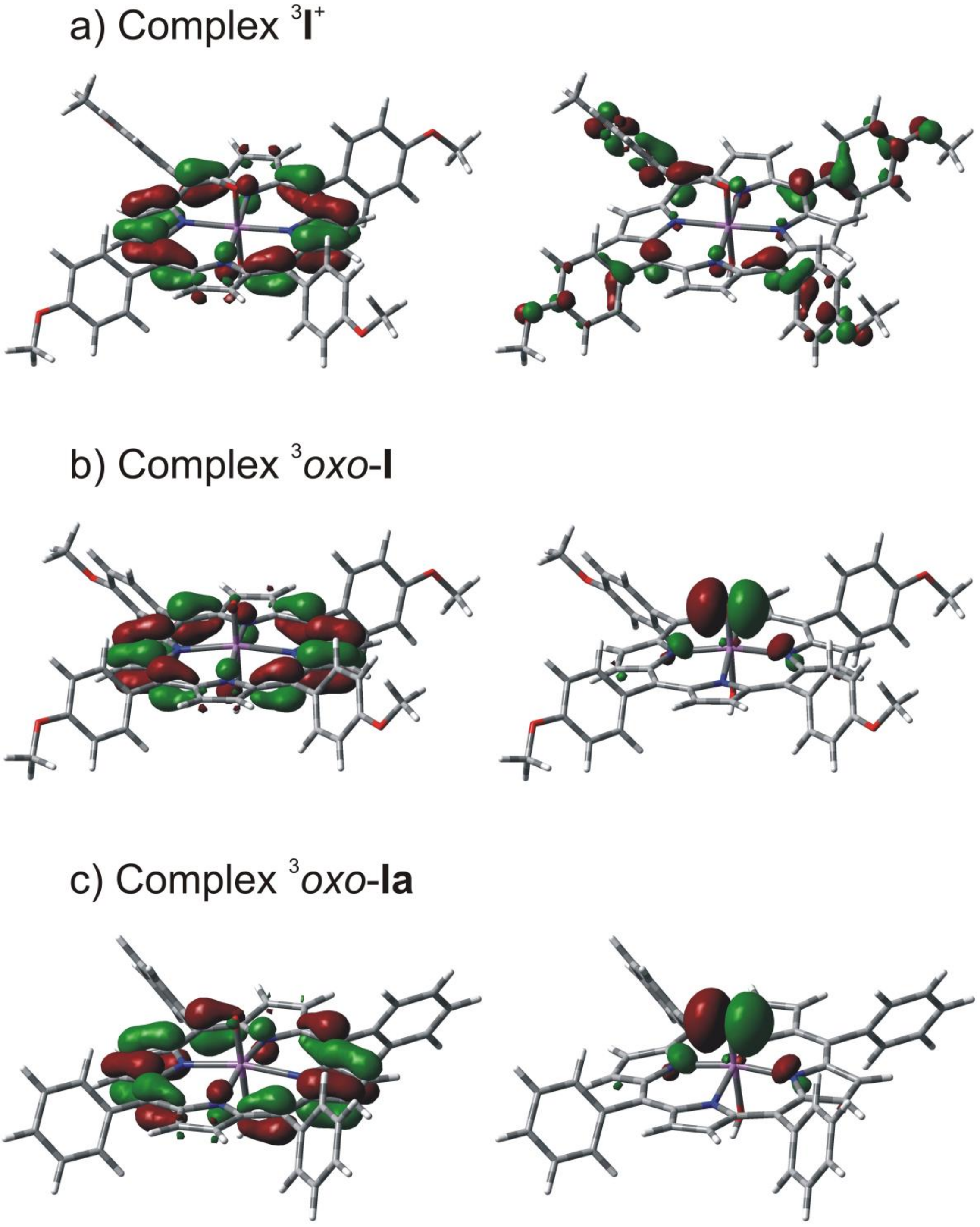


**Figure S3.** Singly Occupied Molecular Orbitals (SOMOs) for the lowest lying triplet state of: a) $^{3}$**I**$^{+}$, b) $^{3}$*oxo*-**I** and c) $^{3}$*oxo*-**Ia** at the UωB97XD/def2SVP level of theory in the gas phase.

## 1.5 Cartesian Coordinates

In all cases, the structures reported below have been optimized at the (U)wB97XD/def2SVP level of theory in the gas phase and it was verified that these have no imaginary frequencies.

**Complex I⁺**

| | | | |
|---|---|---|---|
| N | 0.01739000 | -2.09444200 | -0.02777400 |
| N | 2.08006100 | 0.01779900 | 0.02595900 |
| N | -0.03179500 | 2.07989700 | 0.10242100 |
| C | -2.90640600 | 1.07643200 | -0.01227700 |
| C | -4.26270100 | 0.62070300 | 0.09096500 |
| C | -4.24529100 | -0.74348000 | 0.07730200 |
| C | -2.87819200 | -1.16216000 | -0.03569900 |
| C | -2.43380100 | -2.49748100 | -0.04155300 |
| C | -1.09060200 | -2.90653000 | -0.04998300 |
| C | -0.63432200 | -4.26954600 | -0.07893300 |
| C | 0.72773700 | -4.25133700 | -0.05492400 |
| C | 1.14533100 | -2.87669700 | -0.02797100 |
| C | 2.47785700 | -2.43403400 | 0.00015100 |
| C | 2.88863300 | -1.09169300 | 0.02628400 |
| C | 4.25313700 | -0.64149900 | 0.05031000 |
| C | 4.23934300 | 0.72053600 | 0.07283700 |
| C | 2.86576700 | 1.14464500 | 0.04557100 |
| C | 2.42438500 | 2.47748600 | 0.03690400 |
| C | 1.07863000 | 2.88949100 | 0.03579800 |
| C | 0.62783100 | 4.24679300 | -0.07400500 |
| C | -0.73626500 | 4.23245400 | -0.08391700 |
| C | -1.16024300 | 2.86560200 | 0.01924500 |
| C | -2.49494400 | 2.42198100 | 0.00486000 |
| H | -5.12722600 | 1.27199600 | 0.18847800 |
| H | -5.09231100 | -1.41931600 | 0.15991000 |
| H | -1.28714100 | -5.13762900 | -0.11454900 |
| H | 1.40578200 | -5.10074400 | -0.05764100 |
| H | 5.11781500 | -1.29988700 | 0.05163900 |
| H | 5.09155200 | 1.39387900 | 0.10699800 |
| H | 1.28350200 | 5.10960000 | -0.15674900 |
| H | -1.40792200 | 5.08138800 | -0.17944200 |
| Sb | 0.00061700 | -0.01506400 | 0.00023200 |
| N | -2.09467400 | -0.03270600 | -0.09964400 |
| O | 0.14075700 | 0.07298600 | -1.95996600 |
| O | -0.08166200 | -0.15922300 | 1.96037200 |
| C | -3.56263800 | 3.46154400 | 0.00479700 |
| C | -4.41877900 | 3.61142800 | -1.09887900 |
| C | -3.74023200 | 4.30883200 | 1.10070200 |
| C | -5.41256100 | 4.57510900 | -1.10309900 |
| H | -4.29322100 | 2.96577000 | -1.97143000 |
| C | -4.73992500 | 5.28057100 | 1.11279200 |
| H | -3.09158500 | 4.20461300 | 1.97395900 |
| C | -5.58644100 | 5.42111000 | 0.00523600 |
| H | -6.07484300 | 4.70291300 | -1.96051700 |
| H | -4.85197200 | 5.91540000 | 1.99137000 |
| C | 3.54590100 | -3.47790800 | -0.00146400 |
| C | 4.23936900 | -3.79217900 | 1.16759100 |
| C | 3.87203200 | -4.16440500 | -1.18128500 |
| C | 5.23784200 | -4.76642700 | 1.17629100 |
| H | 3.99508900 | -3.27117700 | 2.09664500 |
| C | 4.86183200 | -5.13341100 | -1.18679400 |
| H | 3.34267000 | -3.92836600 | -2.10764000 |

| | | | |
|---|---|---|---|
| C | 5.55638700 | -5.44590000 | -0.00641300 |
| H | 5.75456100 | -4.98805500 | 2.10980500 |
| H | 5.12555200 | -5.66920500 | -2.09979900 |
| C | 3.46366200 | 3.54688200 | 0.00761900 |
| C | 4.25329900 | 3.74503200 | -1.12656400 |
| C | 3.66606100 | 4.38266300 | 1.11759100 |
| C | 5.22316400 | 4.74614600 | -1.16828200 |
| H | 4.10475500 | 3.11096100 | -2.00413300 |
| C | 4.62779700 | 5.37862500 | 1.09128100 |
| H | 3.06314700 | 4.24122800 | 2.01792700 |
| C | 5.41781700 | 5.57281100 | -0.05413800 |
| H | 5.81420500 | 4.87423800 | -2.07480700 |
| H | 4.79661200 | 6.02689600 | 1.95233100 |
| C | -3.47528900 | -3.56463700 | -0.01189800 |
| C | -4.30337400 | -3.79108500 | -1.11253200 |
| C | -3.64518800 | -4.36566700 | 1.12910200 |
| C | -5.27748000 | -4.78897200 | -1.09375600 |
| H | -4.18383400 | -3.18293400 | -2.01262100 |
| C | -4.61067400 | -5.35752200 | 1.16291100 |
| H | -3.01322400 | -4.19840800 | 2.00470600 |
| C | -5.43784500 | -5.58227000 | 0.04968900 |
| H | -5.89853800 | -4.94075000 | -1.97622300 |
| H | -4.75371200 | -5.97923600 | 2.04788400 |
| O | 6.49614600 | -6.39650200 | -0.10772700 |
| O | 6.32009700 | 6.56112700 | 0.01118200 |
| O | -6.57476700 | 6.32000300 | -0.08711400 |
| O | -6.34056000 | -6.56457500 | 0.17305200 |
| C | 7.23426200 | -6.76553000 | 1.03041100 |
| H | 7.93224100 | -7.54706400 | 0.70936500 |
| H | 7.80986200 | -5.91531800 | 1.43414300 |
| H | 6.58268100 | -7.17096500 | 1.82297400 |
| C | -7.20505400 | -6.85165900 | -0.89811700 |
| H | -6.64724100 | -7.16529100 | -1.79668500 |
| H | -7.84423800 | -7.67891500 | -0.56946800 |
| H | -7.84087700 | -5.98586500 | -1.14939300 |
| C | -6.81274100 | 7.20358100 | 0.98102100 |
| H | -7.65569600 | 7.83579600 | 0.68011300 |
| H | -7.08266900 | 6.66139100 | 1.90295600 |
| H | -5.93712300 | 7.84447900 | 1.17949000 |
| C | 7.14942700 | 6.81806500 | -1.09502900 |
| H | 6.56237300 | 7.10233600 | -1.98466900 |
| H | 7.79527700 | 7.65689400 | -0.81189700 |
| H | 7.78039400 | 5.94691300 | -1.33979200 |
| H | -0.09055700 | 0.71275400 | 2.37257300 |
| H | -0.73165200 | 0.06461400 | -2.37127200 |

## Complex *oxo*-I

| | | | |
|---|---|---|---|
| N | 0.00840400 | -2.12296900 | 0.06328300 |
| N | -2.11657600 | -0.00961900 | -0.01586000 |
| N | 0.00192300 | 2.10750500 | -0.10887300 |
| C | 2.90518700 | 1.11052800 | -0.03717200 |
| C | 4.28017800 | 0.67637700 | -0.15834000 |
| C | 4.28146000 | -0.68572700 | -0.13665700 |
| C | 2.90782800 | -1.11794900 | -0.00142600 |
| C | 2.46327600 | -2.45745300 | 0.02112100 |
| C | 1.12479000 | -2.90327600 | 0.04165500 |
| C | 0.69098400 | -4.28235600 | 0.01627300 |
| C | -0.67139800 | -4.28252000 | -0.00240500 |

| | | | |
|---|---|---|---|
| C | -1.10623900 | -2.90411500 | 0.02604100 |
| C | -2.44638300 | -2.46252600 | -0.00981100 |
| C | -2.89523800 | -1.12588700 | -0.04065100 |
| C | -4.27555800 | -0.69568000 | -0.10144800 |
| C | -4.27782900 | 0.66609700 | -0.12033100 |
| C | -2.89909000 | 1.10312500 | -0.05901600 |
| C | -2.45583800 | 2.44321400 | -0.05242100 |
| C | -1.11601500 | 2.89004400 | -0.05775100 |
| C | -0.68387900 | 4.26583600 | 0.01686100 |
| C | 0.67966400 | 4.26842000 | 0.01816600 |
| C | 1.11758300 | 2.89406800 | -0.05476900 |
| C | 2.45834300 | 2.45076400 | -0.04879000 |
| H | 5.13564900 | 1.33776400 | -0.27054100 |
| H | 5.13768600 | -1.34978100 | -0.22509900 |
| H | 1.35363700 | -5.14402600 | 0.00608500 |
| H | -1.33414800 | -5.14354200 | -0.03855900 |
| H | -5.13481100 | -1.36105400 | -0.12958600 |
| H | -5.13919600 | 1.32681300 | -0.17762200 |
| H | -1.34675000 | 5.12525700 | 0.07927900 |
| H | 1.33874400 | 5.13037200 | 0.08375500 |
| Sb | 0.00231100 | -0.01209300 | 0.26311600 |
| N | 2.12917600 | -0.00337700 | 0.05928000 |
| O | -0.03887300 | 0.09900400 | 2.09552900 |
| O | 0.04653100 | -0.14999200 | -1.73126400 |
| C | 3.51504600 | 3.50575500 | -0.05798100 |
| C | 4.34908300 | 3.69399800 | 1.05552800 |
| C | 3.70471000 | 4.32939800 | -1.16823500 |
| C | 5.33327700 | 4.66997400 | 1.05470000 |
| H | 4.21181100 | 3.06379800 | 1.93734400 |
| C | 4.69195700 | 5.31616900 | -1.18508200 |
| H | 3.07026200 | 4.19556400 | -2.04769500 |
| C | 5.51507800 | 5.49150900 | -0.06762400 |
| H | 5.97943100 | 4.82390500 | 1.92038100 |
| H | 4.80942700 | 5.93342700 | -2.07571900 |
| C | -3.49767500 | -3.52544500 | -0.03295200 |
| C | -4.18571700 | -3.83032200 | -1.20711900 |
| C | -3.81441000 | -4.24512700 | 1.12898000 |
| C | -5.16761000 | -4.82254300 | -1.23881400 |
| H | -3.94831500 | -3.28315200 | -2.12262300 |
| C | -4.78624600 | -5.23396600 | 1.11312900 |
| H | -3.28735800 | -4.01746500 | 2.05854600 |
| C | -5.47315900 | -5.53227800 | -0.07276000 |
| H | -5.67942100 | -5.03205300 | -2.17809900 |
| H | -5.03928700 | -5.79464800 | 2.01439200 |
| C | -3.51339200 | 3.49817100 | -0.02586900 |
| C | -4.28956700 | 3.69920900 | 1.11629800 |
| C | -3.75320200 | 4.31442600 | -1.14161200 |
| C | -5.28070400 | 4.68095000 | 1.16000800 |
| H | -4.11224400 | 3.07778400 | 1.99730400 |
| C | -4.73427800 | 5.29384300 | -1.11399300 |
| H | -3.15907200 | 4.17073600 | -2.04726200 |
| C | -5.50859100 | 5.48685000 | 0.03942200 |
| H | -5.86026800 | 4.80770200 | 2.07436700 |
| H | -4.92799000 | 5.92766300 | -1.98084600 |
| C | 3.52273300 | -3.51117600 | -0.01031300 |
| C | 4.31237900 | -3.76227500 | 1.11185300 |
| C | 3.75392700 | -4.26935600 | -1.16805800 |
| C | 5.30834000 | -4.74046900 | 1.09701900 |
| H | 4.14420800 | -3.18349900 | 2.02319200 |
| C | 4.73934800 | -5.24424300 | -1.19831100 |
| H | 3.14928000 | -4.08237200 | -2.05869300 |
| C | 5.52677900 | -5.48980100 | -0.06383100 |
| H | 5.89955600 | -4.90794800 | 1.99726300 |

| | | | |
|---|---|---|---|
| H | 4.92641600 | -5.83396100 | -2.09716500 |
| O | -6.40122400 | -6.50804800 | 0.00555400 |
| O | -6.43841000 | 6.46154300 | -0.02352900 |
| O | 6.49638200 | 6.41204100 | 0.02046900 |
| O | 6.46013400 | -6.45596400 | -0.18433300 |
| C | -7.12380000 | -6.85515300 | -1.14357200 |
| H | -7.81256500 | -7.65726000 | -0.85192600 |
| H | -7.71143300 | -6.00452900 | -1.53129200 |
| H | -6.46369800 | -7.22709900 | -1.94671900 |
| C | 7.28102900 | -6.75123500 | 0.91237600 |
| H | 6.69552400 | -7.10021700 | 1.78091000 |
| H | 7.95474200 | -7.55571700 | 0.59345300 |
| H | 7.88630200 | -5.87986300 | 1.21800300 |
| C | 6.72956400 | 7.26680200 | -1.06519000 |
| H | 7.55935500 | 7.92307800 | -0.77627500 |
| H | 7.01693300 | 6.70786400 | -1.97296000 |
| H | 5.84664400 | 7.88887700 | -1.29440000 |
| C | -7.24507800 | 6.70749800 | 1.09592700 |
| H | -6.64747800 | 7.01056900 | 1.97334500 |
| H | -7.91753100 | 7.53016200 | 0.82429700 |
| H | -7.85194800 | 5.82555100 | 1.36579000 |
| H | 0.03332000 | 0.74571900 | -2.08605400 |

**Complex $^{3}I^{+}$**

| | | | |
|---|---|---|---|
| N | -0.15986000 | -2.10630500 | 0.05556000 |
| N | -2.07877200 | 0.15357700 | -0.05445300 |
| N | 0.17608800 | 2.08626400 | -0.16066500 |
| C | 2.98344800 | 0.86512100 | -0.07379800 |
| C | 4.26013400 | 0.33981600 | -0.30782900 |
| C | 4.14864900 | -1.04970600 | -0.28137200 |
| C | 2.80487200 | -1.35599400 | -0.03145200 |
| C | 2.25080400 | -2.68866800 | 0.03612900 |
| C | 0.88915400 | -2.99845700 | 0.09515700 |
| C | 0.33795700 | -4.32669800 | 0.19719000 |
| C | -1.01526800 | -4.21696000 | 0.17260500 |
| C | -1.34133000 | -2.81571400 | 0.08997300 |
| C | -2.63257500 | -2.29291200 | 0.00812200 |
| C | -2.97001700 | -0.89257200 | -0.09036900 |
| C | -4.26970100 | -0.37309600 | -0.18155300 |
| C | -4.15911400 | 1.01384300 | -0.21819000 |
| C | -2.79518500 | 1.32769700 | -0.11705600 |
| C | -2.24157600 | 2.65800800 | -0.06900500 |
| C | -0.87864500 | 2.97164800 | -0.04755600 |
| C | -0.33603200 | 4.28776800 | 0.16200600 |
| C | 1.01889200 | 4.18155700 | 0.17761800 |
| C | 1.35444100 | 2.79644400 | -0.02441700 |
| C | 2.65066800 | 2.27223900 | -0.03285700 |
| H | 5.15816900 | 0.92185600 | -0.49382900 |
| H | 4.94067200 | -1.77439800 | -0.44566000 |
| H | 0.92488200 | -5.23715700 | 0.27729300 |
| H | -1.74516700 | -5.02101200 | 0.20418500 |
| H | -5.18305400 | -0.95990300 | -0.20469800 |
| H | -4.96691400 | 1.73383100 | -0.30907600 |
| H | -0.92891600 | 5.18461700 | 0.31806200 |
| H | 1.74054800 | 4.97459600 | 0.35164600 |
| Sb | 0.00005200 | -0.01834800 | -0.01372700 |
| N | 2.09790200 | -0.17866100 | 0.09726300 |
| O | -0.13188200 | 0.12591600 | 1.94326100 |

| | | | |
|---|---|---|---|
| O | 0.08411800 | -0.21164900 | -1.96926100 |
| C | 3.78474600 | 3.21965500 | -0.00611100 |
| C | 4.77368200 | 3.13475100 | 0.99381800 |
| C | 3.91226500 | 4.22249000 | -0.97497800 |
| C | 5.82871100 | 4.02644100 | 1.02913600 |
| H | 4.69551100 | 2.36598300 | 1.76548400 |
| C | 4.97977700 | 5.11640100 | -0.96140100 |
| H | 3.17304000 | 4.29413500 | -1.77615200 |
| C | 5.94779000 | 5.02782600 | 0.04850500 |
| H | 6.58606200 | 3.97781000 | 1.81273000 |
| H | 5.05137300 | 5.86965700 | -1.74548600 |
| C | -3.76644500 | -3.25063900 | 0.02772300 |
| C | -4.58251900 | -3.41546200 | -1.09507000 |
| C | -4.04224500 | -4.01574300 | 1.17383800 |
| C | -5.64034000 | -4.32219900 | -1.09312800 |
| H | -4.38075600 | -2.83549100 | -1.99862800 |
| C | -5.09763200 | -4.91116200 | 1.19219900 |
| H | -3.42542700 | -3.89176300 | 2.06707400 |
| C | -5.90785700 | -5.07786100 | 0.05656500 |
| H | -6.24432700 | -4.43356700 | -1.99326900 |
| H | -5.32575900 | -5.49963100 | 2.08199500 |
| C | -3.20448200 | 3.78110700 | -0.02403300 |
| C | -4.13904400 | 3.88721700 | 1.01306500 |
| C | -3.19869600 | 4.77660100 | -1.01852700 |
| C | -5.03476100 | 4.95089100 | 1.07485700 |
| H | -4.15407200 | 3.13347200 | 1.80336300 |
| C | -4.09463600 | 5.82956300 | -0.97774400 |
| H | -2.49063300 | 4.70664300 | -1.84752100 |
| C | -5.02227200 | 5.93227200 | 0.07323800 |
| H | -5.73318100 | 5.00703100 | 1.90934200 |
| H | -4.10685600 | 6.59427500 | -1.75558200 |
| C | 3.21835600 | -3.80863200 | 0.00322300 |
| C | 4.20494500 | -3.93523500 | 0.98799700 |
| C | 3.17710100 | -4.76915200 | -1.02438500 |
| C | 5.11344900 | -4.98994200 | 0.97283400 |
| H | 4.25590500 | -3.20322700 | 1.79715700 |
| C | 4.08415700 | -5.81284600 | -1.05990900 |
| H | 2.43322400 | -4.67663000 | -1.81903000 |
| C | 5.06091400 | -5.93978400 | -0.05739800 |
| H | 5.85399100 | -5.06361000 | 1.76878700 |
| H | 4.06757400 | -6.55080100 | -1.86310500 |
| O | -6.90036100 | -5.97024200 | 0.16487600 |
| O | -5.84377000 | 6.98693700 | 0.03420200 |
| O | 7.00558100 | 5.83761400 | 0.15851400 |
| O | 5.88953300 | -6.98387600 | -0.16922300 |
| C | -7.75662000 | -6.19389200 | -0.92826500 |
| H | -8.47929800 | -6.95256500 | -0.60727900 |
| H | -8.30107000 | -5.27705100 | -1.21066000 |
| H | -7.20443700 | -6.57308700 | -1.80465200 |
| C | 6.90090500 | -7.17732900 | 0.78975400 |
| H | 6.47926600 | -7.33070000 | 1.79728300 |
| H | 7.44482100 | -8.08008900 | 0.48996500 |
| H | 7.60298300 | -6.32705700 | 0.81412000 |
| C | 7.19674100 | 6.86755100 | -0.78164400 |
| H | 8.11221800 | 7.39200100 | -0.48599300 |
| H | 7.32638500 | 6.46565900 | -1.80040800 |
| H | 6.35601300 | 7.58129300 | -0.77519400 |
| C | -6.80783100 | 7.15811400 | 1.04515000 |
| H | -6.33826200 | 7.27628200 | 2.03603100 |
| H | -7.35655000 | 8.07410200 | 0.79915100 |
| H | -7.51565800 | 6.31291600 | 1.07579500 |
| H | 0.17201600 | 0.64876800 | -2.39596500 |
| H | 0.74336000 | 0.06066400 | 2.34392800 |

**Complex $^{3}$*oxo*-I**

| | | | |
|---|---|---|---|
| N | -0.17199200 | -2.13096200 | 0.06787300 |
| N | -2.11850300 | 0.16608500 | -0.06629800 |
| N | 0.18244100 | 2.11618600 | -0.19837000 |
| C | 3.00079800 | 0.85024600 | -0.13356400 |
| C | 4.29170800 | 0.32133400 | -0.35970500 |
| C | 4.17698900 | -1.06349600 | -0.32253100 |
| C | 2.81736300 | -1.35979800 | -0.07466800 |
| C | 2.23623700 | -2.68447900 | 0.01078800 |
| C | 0.87370200 | -3.01103700 | 0.07753600 |
| C | 0.31870200 | -4.35047600 | 0.13725600 |
| C | -1.03375100 | -4.23518300 | 0.12033400 |
| C | -1.35230800 | -2.82082200 | 0.07458900 |
| C | -2.64006700 | -2.27416700 | -0.00740600 |
| C | -2.99768200 | -0.87350900 | -0.10972600 |
| C | -4.30559500 | -0.35141700 | -0.23359800 |
| C | -4.18883600 | 1.03269200 | -0.27303100 |
| C | -2.81310500 | 1.33725900 | -0.15204800 |
| C | -2.23105600 | 2.66260100 | -0.10763800 |
| C | -0.86684700 | 2.99278900 | -0.09830900 |
| C | -0.31870500 | 4.32288400 | 0.07355600 |
| C | 1.03502200 | 4.21135600 | 0.08154500 |
| C | 1.36019400 | 2.80929900 | -0.08707900 |
| C | 2.65088400 | 2.25792100 | -0.09295600 |
| H | 5.19222300 | 0.90079700 | -0.54516700 |
| H | 4.96864700 | -1.79228700 | -0.47390700 |
| H | 0.90166700 | -5.26649700 | 0.17981500 |
| H | -1.76613500 | -5.03816200 | 0.13034100 |
| H | -5.22126300 | -0.93443200 | -0.28022900 |
| H | -4.99283700 | 1.75574600 | -0.38013200 |
| H | -0.90537100 | 5.22828300 | 0.20449400 |
| H | 1.76014600 | 5.00828900 | 0.22257900 |
| Sb | 0.00151700 | -0.00921300 | 0.22246700 |
| N | 2.13156800 | -0.18664200 | 0.03308800 |
| O | -0.04177600 | 0.16316000 | 2.05147600 |
| O | 0.04430000 | -0.19889500 | -1.76635100 |
| C | 3.79435900 | 3.20425200 | -0.06221300 |
| C | 4.73669800 | 3.15503400 | 0.98055300 |
| C | 3.97148600 | 4.16183200 | -1.06373400 |
| C | 5.80003600 | 4.04021200 | 1.02296600 |
| H | 4.61668800 | 2.41391400 | 1.77357700 |
| C | 5.04506700 | 5.05273400 | -1.04013200 |
| H | 3.26159200 | 4.20667500 | -1.89287900 |
| C | 5.96700400 | 4.99819000 | 0.01052400 |
| H | 6.52496900 | 4.01562400 | 1.83801900 |
| H | 5.15149300 | 5.77599600 | -1.84847100 |
| C | -3.77671500 | -3.23644100 | -0.00294500 |
| C | -4.54240700 | -3.44549000 | -1.15098200 |
| C | -4.10274200 | -3.95785100 | 1.15581300 |
| C | -5.60228400 | -4.35271200 | -1.16111500 |
| H | -4.30258400 | -2.89287200 | -2.06223100 |
| C | -5.15682100 | -4.85824300 | 1.16142900 |
| H | -3.51987900 | -3.80007100 | 2.06624800 |
| C | -5.91647900 | -5.06606000 | 0.00107900 |
| H | -6.16945300 | -4.49389300 | -2.08108600 |
| H | -5.41915500 | -5.41748000 | 2.06084600 |
| C | -3.19674400 | 3.79122100 | -0.05262300 |
| C | -4.07688700 | 3.92345700 | 1.02487100 |
| C | -3.25055800 | 4.75349900 | -1.07383700 |
| C | -4.97929000 | 4.98330000 | 1.09986900 |
| H | -4.04846500 | 3.18639300 | 1.83033400 |

| | | | |
|---|---|---|---|
| C | -4.14986500 | 5.80661100 | -1.01760000 |
| H | -2.57998100 | 4.66263300 | -1.93146200 |
| C | -5.02242700 | 5.93382500 | 0.07302600 |
| H | -5.63849600 | 5.05574000 | 1.96473200 |
| H | -4.20266200 | 6.55038200 | -1.81415300 |
| C | 3.20492800 | -3.81249000 | -0.01551100 |
| C | 4.13354900 | -3.97703600 | 1.01506200 |
| C | 3.22266600 | -4.73055100 | -1.07775000 |
| C | 5.04709600 | -5.03045200 | 1.00817600 |
| H | 4.13843900 | -3.27006400 | 1.84765300 |
| C | 4.13138700 | -5.77681200 | -1.10221100 |
| H | 2.51693600 | -4.60839200 | -1.90262300 |
| C | 5.05129000 | -5.93948900 | -0.05617700 |
| H | 5.74617200 | -5.12977800 | 1.83841000 |
| H | 4.15561300 | -6.48707400 | -1.93011400 |
| O | -6.91910300 | -5.96215900 | 0.09797000 |
| O | -5.85880500 | 6.98970300 | 0.04649000 |
| O | 7.03316600 | 5.81222700 | 0.13198900 |
| O | 5.89371500 | -6.98604000 | -0.16107800 |
| C | -7.71915200 | -6.21534600 | -1.02446300 |
| H | -8.45981300 | -6.96466300 | -0.72002700 |
| H | -8.24889700 | -5.30792900 | -1.36341300 |
| H | -7.12927900 | -6.61896900 | -1.86595800 |
| C | 6.84368000 | -7.20205400 | 0.84698600 |
| H | 6.36780600 | -7.38681800 | 1.82580000 |
| H | 7.41307500 | -8.09309100 | 0.55632400 |
| H | 7.53929900 | -6.35029300 | 0.94354300 |
| C | 7.25797900 | 6.79414000 | -0.84296100 |
| H | 8.16476500 | 7.33296600 | -0.54305100 |
| H | 7.42030400 | 6.35080400 | -1.84085000 |
| H | 6.42051900 | 7.51098500 | -0.90257500 |
| C | -6.76160700 | 7.17335700 | 1.10351500 |
| H | -6.24121600 | 7.31618200 | 2.06648600 |
| H | -7.33493900 | 8.07926400 | 0.87286000 |
| H | -7.45989600 | 6.32360100 | 1.19770800 |
| H | 0.12467100 | 0.68390200 | -2.14306000 |

## Complex $^{3}$*oxo*-Ia

| | | | |
|---|---|---|---|
| N | 2.13168300 | -0.01373000 | -0.01402900 |
| N | 0.01390200 | 2.13468900 | -0.13863500 |
| N | -2.13177200 | 0.01423600 | -0.01417900 |
| C | -1.13574200 | -2.91115600 | 0.03595000 |
| C | -0.70695600 | -4.29270100 | 0.15374800 |
| C | 0.65045800 | -4.30159400 | 0.15415600 |
| C | 1.09747400 | -2.92582500 | 0.03637700 |
| C | 2.42891400 | -2.49243300 | -0.02617000 |
| C | 2.90832800 | -1.12743900 | -0.11593500 |
| C | 4.24661400 | -0.72503700 | -0.32998100 |
| C | 4.25542300 | 0.66473700 | -0.34656000 |
| C | 2.92236200 | 1.08996000 | -0.14254100 |
| C | 2.46314300 | 2.46156500 | -0.07461300 |
| C | 1.13610000 | 2.91508300 | -0.04440300 |
| C | 0.70733500 | 4.29020000 | 0.11799300 |
| C | -0.65112700 | 4.29913200 | 0.11811500 |
| C | -1.09799500 | 2.92976200 | -0.04410700 |
| C | -2.43088500 | 2.49360100 | -0.07398000 |
| C | -2.90799200 | 1.12811900 | -0.14233600 |
| C | -4.24649200 | 0.72029400 | -0.34638000 |

| | | | |
|---|---|---|---|
| C | -4.25589700 | -0.66946900 | -0.32988200 |
| C | -2.92293500 | -1.08926600 | -0.11617200 |
| C | -2.46134100 | -2.46040400 | -0.02708200 |
| H | -1.37318800 | -5.14720700 | 0.23817000 |
| H | 1.30511900 | -5.16493400 | 0.23894300 |
| H | 5.09567900 | -1.38953200 | -0.46653000 |
| H | 5.11324900 | 1.31444500 | -0.49897700 |
| H | 1.37226800 | 5.14118500 | 0.24031800 |
| H | -1.30451100 | 5.15897100 | 0.24057400 |
| H | -5.09558100 | 1.38141300 | -0.49870400 |
| H | -5.11377100 | -1.32249700 | -0.46659500 |
| Sb | -0.00009400 | -0.00708000 | 0.21978600 |
| N | -0.01400000 | -2.13165100 | -0.00828700 |
| O | -0.00076600 | -0.12505700 | -1.77416000 |
| H | 0.00578100 | 0.77184100 | -2.12506000 |
| O | 0.00047600 | 0.08573600 | 2.05472900 |
| C | -3.52967300 | -3.49802900 | -0.02117800 |
| C | -4.42381400 | -3.58196400 | 1.05422700 |
| C | -3.66089400 | -4.39490300 | -1.08847400 |
| C | -5.42055100 | -4.55434300 | 1.06625800 |
| H | -4.32595200 | -2.88131200 | 1.88636700 |
| C | -4.66488000 | -5.36142100 | -1.07830100 |
| H | -2.97397400 | -4.32328700 | -1.93490600 |
| C | -5.54447400 | -5.44510300 | -0.00011800 |
| H | -6.10480600 | -4.61701900 | 1.91504900 |
| H | -4.76138800 | -6.05068400 | -1.91987800 |
| H | -6.32936600 | -6.20442400 | 0.00890500 |
| C | 3.53219100 | 3.49678800 | -0.03590100 |
| C | 3.66751300 | 4.42757200 | -1.07366800 |
| C | 4.42309400 | 3.54810600 | 1.04481200 |
| C | 4.67145200 | 5.39308700 | -1.03057700 |
| H | 2.98406500 | 4.38381200 | -1.92479600 |
| C | 5.41914400 | 4.51988300 | 1.09066800 |
| H | 4.32169600 | 2.82260400 | 1.85483300 |
| C | 5.54714200 | 5.44325300 | 0.05279200 |
| H | 4.77113400 | 6.10777600 | -1.85027100 |
| H | 6.09981700 | 4.55661900 | 1.94380800 |
| H | 6.33201900 | 6.20179700 | 0.08812700 |
| C | -3.48649800 | 3.54250600 | -0.03428000 |
| C | -3.61008700 | 4.47610200 | -1.07102600 |
| C | -4.37661300 | 3.60400500 | 1.04653500 |
| C | -4.60164400 | 5.45426400 | -1.02673400 |
| H | -2.92746600 | 4.42453200 | -1.92238200 |
| C | -5.36020400 | 4.58832700 | 1.09359700 |
| H | -4.28448600 | 2.87641600 | 1.85579800 |
| C | -5.47652700 | 5.51441900 | 0.05677500 |
| H | -4.69225500 | 6.17106700 | -1.84564500 |
| H | -6.04027700 | 4.63272500 | 1.94685400 |
| H | -6.25168600 | 6.28284900 | 0.09302000 |
| C | 3.48396700 | -3.54354900 | -0.01950100 |
| C | 3.60373300 | -4.44355600 | -1.08560400 |
| C | 4.37732900 | -3.63711400 | 1.05571200 |
| C | 4.59558000 | -5.42250000 | -1.07427100 |
| H | 2.91770500 | -4.36444400 | -1.93208800 |
| C | 5.36184300 | -4.62185600 | 1.06892200 |
| H | 4.28856000 | -2.93409200 | 1.88687600 |
| C | 5.47434800 | -5.51563800 | 0.00380200 |
| H | 4.68322600 | -6.11412100 | -1.91488800 |
| H | 6.04547700 | -4.69185100 | 1.91764100 |
| H | 6.24974200 | -6.28464200 | 0.01368000 |

**Complex I•**

| | | | |
|---|---|---|---|
| N | 1.97822500 | 0.66572500 | -0.02944300 |
| N | 0.66884900 | -1.98452000 | -0.00515900 |
| N | -1.97835600 | -0.66551400 | -0.11382100 |
| C | -1.98633700 | 2.39135600 | -0.03375200 |
| C | -2.00992100 | 3.80012900 | -0.11208700 |
| C | -0.69635800 | 4.24225700 | -0.09678300 |
| C | 0.13516400 | 3.10516000 | -0.00963100 |
| C | 1.56512800 | 3.10970800 | 0.01367300 |
| C | 2.37619300 | 1.98900700 | 0.01583300 |
| C | 3.82397600 | 2.00093900 | 0.08821600 |
| C | 4.25564500 | 0.71806100 | 0.08148200 |
| C | 3.09544800 | -0.14681000 | 0.00433000 |
| C | 3.12588100 | -1.53019200 | -0.01309000 |
| C | 1.98453500 | -2.38658500 | -0.03991700 |
| C | 2.01047100 | -3.79865400 | -0.11615200 |
| C | 0.69996500 | -4.24010000 | -0.12443400 |
| C | -0.13466100 | -3.10074700 | -0.05388600 |
| C | -1.56362700 | -3.10957000 | -0.04270300 |
| C | -2.37692000 | -1.99147800 | -0.04976000 |
| C | -3.82356600 | -2.00348900 | 0.03653800 |
| C | -4.25712900 | -0.72133600 | 0.03760200 |
| C | -3.09929500 | 0.14752400 | -0.04723400 |
| C | -3.13026800 | 1.52970100 | -0.03326400 |
| H | -2.91002700 | 4.40490900 | -0.18402600 |
| H | -0.34310500 | 5.26833400 | -0.15519100 |
| H | 4.42533600 | 2.90428700 | 0.15125400 |
| H | 5.28083200 | 0.36110700 | 0.13761600 |
| H | 2.91290900 | -4.40190100 | -0.16816200 |
| H | 0.34725100 | -5.26618900 | -0.18473300 |
| H | -4.42382400 | -2.90706300 | 0.10798500 |
| H | -5.28226000 | -0.36736900 | 0.11153500 |
| Sb | 0.01037000 | -0.00506500 | -0.02611900 |
| N | -0.66976600 | 1.98650600 | 0.03482800 |
| O | -0.02203000 | -0.08371200 | 1.94939000 |
| O | 0.08358400 | 0.05559000 | -2.00045400 |
| C | -4.46460100 | 2.19883500 | -0.01610700 |
| C | -4.91174500 | 2.88605800 | 1.12364100 |
| C | -5.30302300 | 2.16643400 | -1.13050900 |
| C | -6.15004100 | 3.50805700 | 1.14680800 |
| H | -4.27080400 | 2.92658100 | 2.00746400 |
| C | -6.55459200 | 2.78634600 | -1.12414900 |
| H | -4.97116000 | 1.64504600 | -2.03157300 |
| C | -6.98542900 | 3.46369300 | 0.02065100 |
| H | -6.50276700 | 4.03775800 | 2.03326300 |
| H | -7.17536100 | 2.73681400 | -2.01874800 |
| C | 4.46079500 | -2.19901800 | -0.00486200 |
| C | 5.28065900 | -2.18382800 | -1.13308600 |
| C | 4.92356400 | -2.86699700 | 1.13958200 |
| C | 6.53176400 | -2.80473400 | -1.13551100 |
| H | 4.93392000 | -1.67587900 | -2.03622300 |
| C | 6.16205300 | -3.48957000 | 1.15323700 |
| H | 4.29516000 | -2.89132600 | 2.03298100 |
| C | 6.97951900 | -3.46384400 | 0.01358300 |
| H | 7.13922900 | -2.76963400 | -2.03988900 |
| H | 6.52826600 | -4.00543400 | 2.04239500 |
| C | -2.22158200 | -4.44965100 | -0.01247300 |
| C | -2.17151400 | -5.24490300 | 1.13343100 |
| C | -2.90448300 | -4.94869200 | -1.13161800 |
| C | -2.78264300 | -6.49874200 | 1.17949000 |
| H | -1.64319200 | -4.87488200 | 2.01530100 |

| | | | |
|---|---|---|---|
| C | -3.51912100 | -6.19189200 | -1.10215400 |
| H | -2.94861000 | -4.34587900 | -2.04184300 |
| C | -3.46368400 | -6.97992800 | 0.05593600 |
| H | -2.72278900 | -7.08434400 | 2.09691500 |
| H | -4.04914800 | -6.58394900 | -1.97172700 |
| C | 2.22583600 | 4.44865600 | 0.02045300 |
| C | 2.18942700 | 5.26361800 | 1.15253900 |
| C | 2.89652300 | 4.92745700 | -1.11517600 |
| C | 2.80292800 | 6.51727600 | 1.17075500 |
| H | 1.67036900 | 4.91103800 | 2.04700100 |
| C | 3.51306900 | 6.16983400 | -1.11344500 |
| H | 2.92866700 | 4.30813600 | -2.01470200 |
| C | 3.47209800 | 6.97771700 | 0.03164600 |
| H | 2.75418300 | 7.11855400 | 2.07864300 |
| H | 4.03414100 | 6.54594300 | -1.99538800 |
| O | 8.16783900 | -4.09532600 | 0.11811400 |
| O | -4.08797400 | -8.17487200 | -0.00472900 |
| O | -8.17198800 | 4.09616100 | 0.13350300 |
| O | 4.09781200 | 8.17027200 | -0.05642200 |
| C | 9.03039500 | -4.10882700 | -0.98546500 |
| H | 9.92299800 | -4.66876900 | -0.68094400 |
| H | 8.57476300 | -4.61150800 | -1.85666100 |
| H | 9.33368500 | -3.08933000 | -1.28214600 |
| C | 4.09112000 | 9.02610400 | 1.05251800 |
| H | 4.58623300 | 8.56889400 | 1.92722800 |
| H | 4.64836300 | 9.92452000 | 0.76045700 |
| H | 3.06579700 | 9.32025600 | 1.33798800 |
| C | -9.05190700 | 4.09197100 | -0.95660600 |
| H | -9.93987200 | 4.65632800 | -0.64689100 |
| H | -8.61015100 | 4.58109100 | -1.84249700 |
| H | -9.35912700 | 3.06776800 | -1.23214400 |
| C | -4.06512200 | -9.01259000 | 1.11792300 |
| H | -4.55113000 | -8.54270800 | 1.99098600 |
| H | -4.62295900 | -9.91728100 | 0.84715600 |
| H | -3.03549500 | -9.29887600 | 1.39560500 |
| H | -0.77391200 | -0.21326900 | -2.34967800 |
| H | -0.33341900 | 0.76394900 | 2.28735000 |

# 2) Experimental Details

## 2.1 General information

Dimethyl maleate (**2a**) and dimethyl fumarate (**2b**) were commercially available and used as received, while tetrahydrofuran (THF, **1a**), heptaldehyde (**1b**) and cyclohexane (**1c**) were distilled prior to use. 2-Cyclohexylidenemalononitrile (**2c**) and 2-benzylidenemalononitrile (**2d**) were synthesized according to a published procedure.[S2] Complexes **I**$^+$ and **Ia**$^+$ were synthesized as previously reported.[S3] Acetonitrile and water (HPLC purity grade) employed for photochemical reactions were used as received. A NaOH stock solution in HPLC-grade water ($4\times10^{-3}$ M) was prepared and, when required, the proper amount of solution was added to the reaction mixture, as detailed in the main text.

Reactions were monitored by gas chromatographic (GC-FID) analyses (HP-5 capillary column). The GC oven temperature was held at 80 °C for 2 min, increased to 250 °C by a temperature ramp of 10 $°C\cdot min^{-1}$ and held for 5 min. Products **3-7** were quantified via calibration curves in the presence of *n*-dodecane (1 $\mu L\cdot mL^{-1}$) as internal standard by comparison with authentic samples. The conversion degree of the employed olefins **2** was determined in the same way.

GC-MS analyses were carried out using a Thermo Scientific DSQII single quadrupole GC-MS system. A Restek Rtx-5MS (30 m × 0.25 mm × 0.25 μm) capillary column was used for analytes separation with helium as carrier gas at 1 $mL\cdot min^{-1}$. The injection in the GC system was performed in split mode and the injector temperature was 250 °C. The GC oven temperature was held at 80 °C for 2 min, increased to 220 °C by a temperature ramp of 10 $°C\cdot min^{-1}$ and held for 10 min. The transfer line temperature was 250 °C and the ion source temperature 250 °C. Mass spectral analyses were carried out in full scan mode.

Irradiation was carried out with different light sources:

- At 366 nm: 10×15 W phosphor-coated lamps Hg-lamps (emission centered at $\lambda_{em}$=366 nm);
- At 405 nm: 1 W LED (for *Method B*) or 18 W Evoluchem LED lamp (for *Method A*);
- At 455 nm: 1 W LED (for *Method B*) or 18 W Evoluchem LED lamp (for *Method A*);
- Medium-pressure Na lamp (emission centered at $\lambda_{em}$ = 589 nm);
- Solar simulator: 1.5 kW Xe lamp, 500 $W\cdot m^{-2}$.

**General procedure for the optimization of reaction conditions.**

Experiments reported in this work were performed either in a one-dram vial (*method A*) or in a 1 mm cuvette (*method B*) as the reaction vessel. Cuvettes were washed before use with a $KMnO_4/H_2SO_4$ solution and then distilled water; finally, they were rinsed with acetone and oven-dried (50°C).

- *Method A* (scale: 0.05 mmol): in a typical experiment, dimethyl fumarate **2b** (7.2 mg, 0.05 M, 1 equiv.), tetrahydrofuran **1a** (40 μL, 0.5 M, 10 equiv.) and *n*-dodecane (internal std, 1 μl) were dissolved in 1 mL of a $MeCN/H_2O$ 95:5 solution $5\cdot10^{-4}$ M both in Complex **I**$^+$ and NaOH. The resulting green solution was flushed with $N_2$ and finally irradiated with the selected irradiation system for 48 hours.

- *Method B* (scale: 0.015 mmol): 300 µL of the solution prepared as detailed in *method A* were transferred into a 1 mm cuvette, flushed with $N_2$ and finally irradiated with the selected irradiation system for 48 hours (see Figure S4).

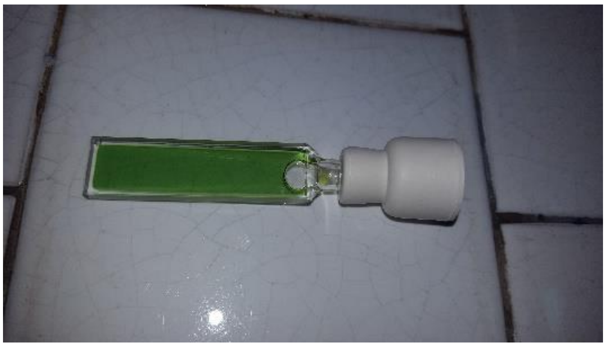

**Figure S4**. Example of a solution prepared according to *method B* prior to irradiation.

After irradiation, the yield of the products was determined by GC-FID analyses through calibration curves obtained with authentic samples of **3-7**, using *n*-dodecane as the internal standard. Yields were double-checked via $^1$H-NMR using dibromomethane as external standard. Identification of the products was possible by comparing both GC-FID chromatograms and $^1$H-NMR spectra with those of authentic samples of **3**-**7**.

**Synthesis of dimethyl 2-(tetrahydrofuran-2-yl)succinate (3).**[S4]

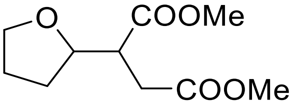


- from dimethyl maleate **2a** (6.3 µL, 0.05 M, 1.0 equiv.) and tetrahydrofuran **1a** (40 µL, 0.5 M, 10.0 equiv.).
- from dimethyl fumarate **2b** (7.2 mg, 0.05 M, 1.0 equiv.) and tetrahydrofuran **1a** (40 µL, 0.5 M, 10 equiv.).

**Synthesis of 2-(1-(tetrahydrofuran-2-yl)cyclohexyl)malononitrile (4).**[S5]

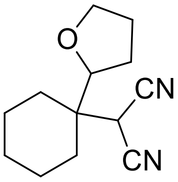


- from 2-cyclohexylidenemalononitrile **2c** (7.3 mg, 0.05 M, 1.0 equiv.) and tetrahydrofuran **1a** (40 µL, 0.5 M, 10.0 equiv.).

**Synthesis of 2-(phenyl(tetrahydrofuran-2-yl)methyl)malononitrile (5).**[S6]

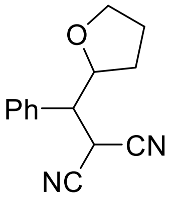


- from 2-benzylidenemalononitrile **2d** (7.5 mg, 0.05 M, 1.0 equiv.) and tetrahydrofuran **1a** (40 μL, 0.5 M, 10.0 equiv.).

**Synthesis of 2-(phenyl(tetrahydrofuran-2-yl)methyl)malononitrile (6).**[S5]

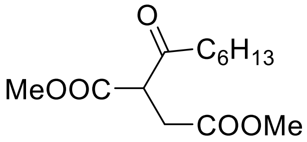


- from dimethylfumarate **2b** (7.2 mg, 0.05 M, 1.0 equiv.) and heptaldehyde **1b** (7 μL, 0.05 M, 1.0 equiv.).

**Synthesis of 2-(2-oxo-1-phenyloctyl)malononitrile (7).**[S6]

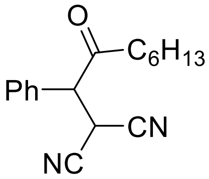


- from 2-benzylidenemalononitrile **2d** (7.5 mg, 0.05 M, 1.0 equiv.) and heptaldehyde **1b** (7 μL, 0.05 M, 1.0 equiv.).

## 2.2 Chemical quenching experiments

*Effect of a radical scavenger on the reaction*: to a solution prepared according to the general procedure *(Method B)*, TEMPO (7.8 mg, 0.05 M, 1 equiv.) was added. 300 µL of the freshly prepared solution were transferred into a 1 mm cuvette, sparged with $N_2$ and finally irradiated for 24 hours with a 1 W LED at 405 nm. GC analysis revealed that the reaction was completely inhibited.

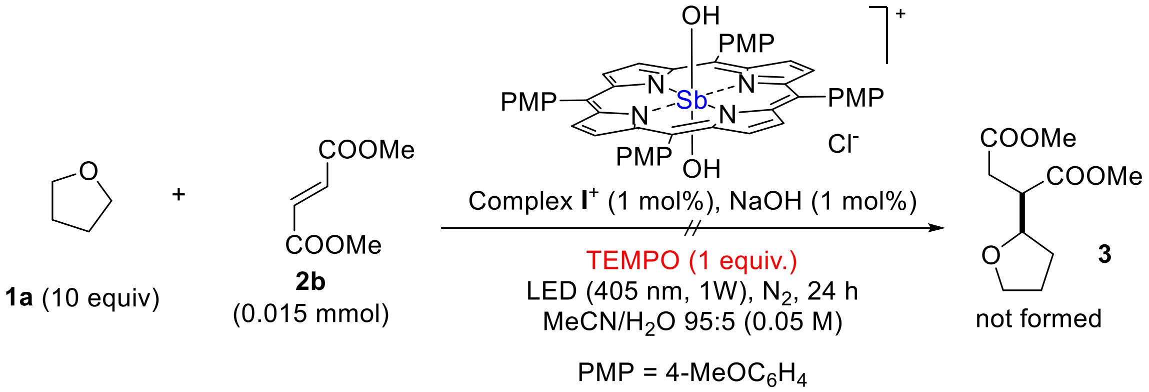


**Figure S5**. Experiment in the presence of the radical scavenger TEMPO ((2,2,6,6-tetramethylpiperidin-1-yl)oxyl).

## 2.3 Deuterium labelling experiments

**Cross-over experiment**

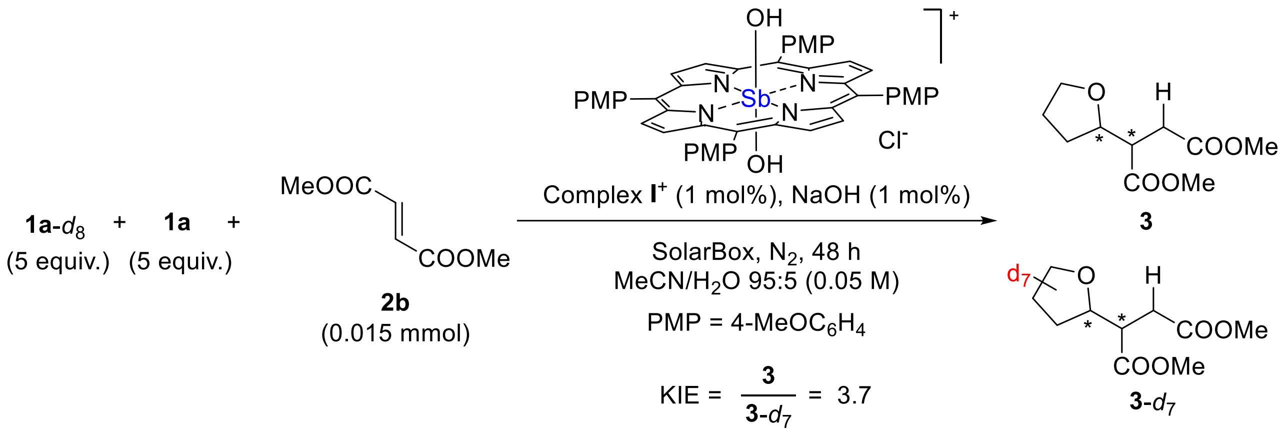


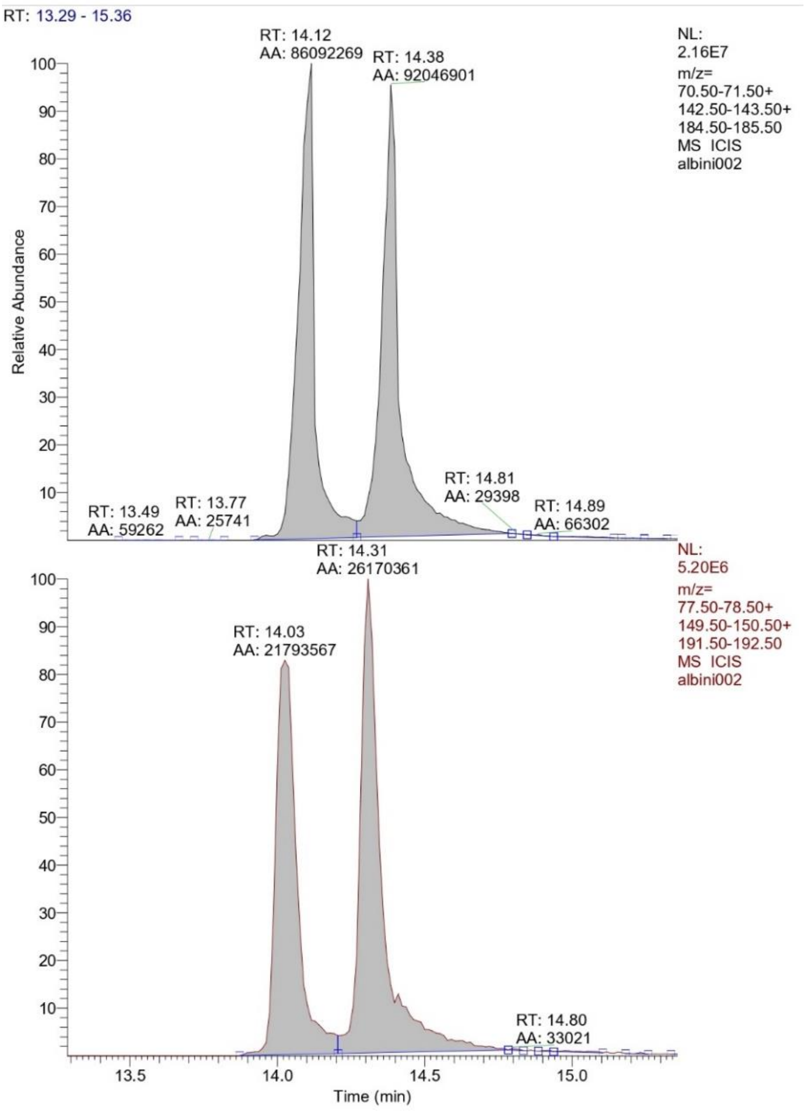


**Figure S6**. Cross-over experiment in the presence of a mixture of **1a** and **1a**-$d_8$. Upper part: scheme of the experiment; lower part: particular of the GC-MS chromatogram of the reaction crude. The KIE value has been determined as the ratio between the area of **3** and **3**-$d_7$.

## Experiment in deuterated acetonitrile

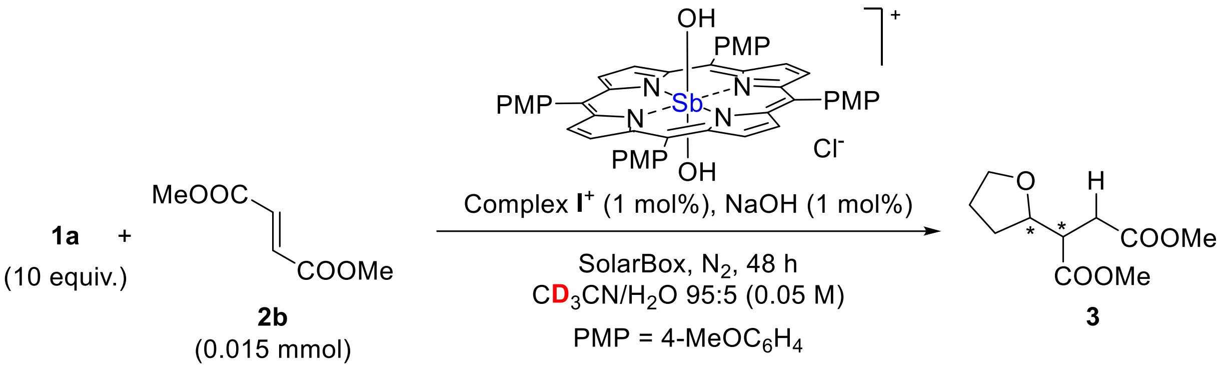


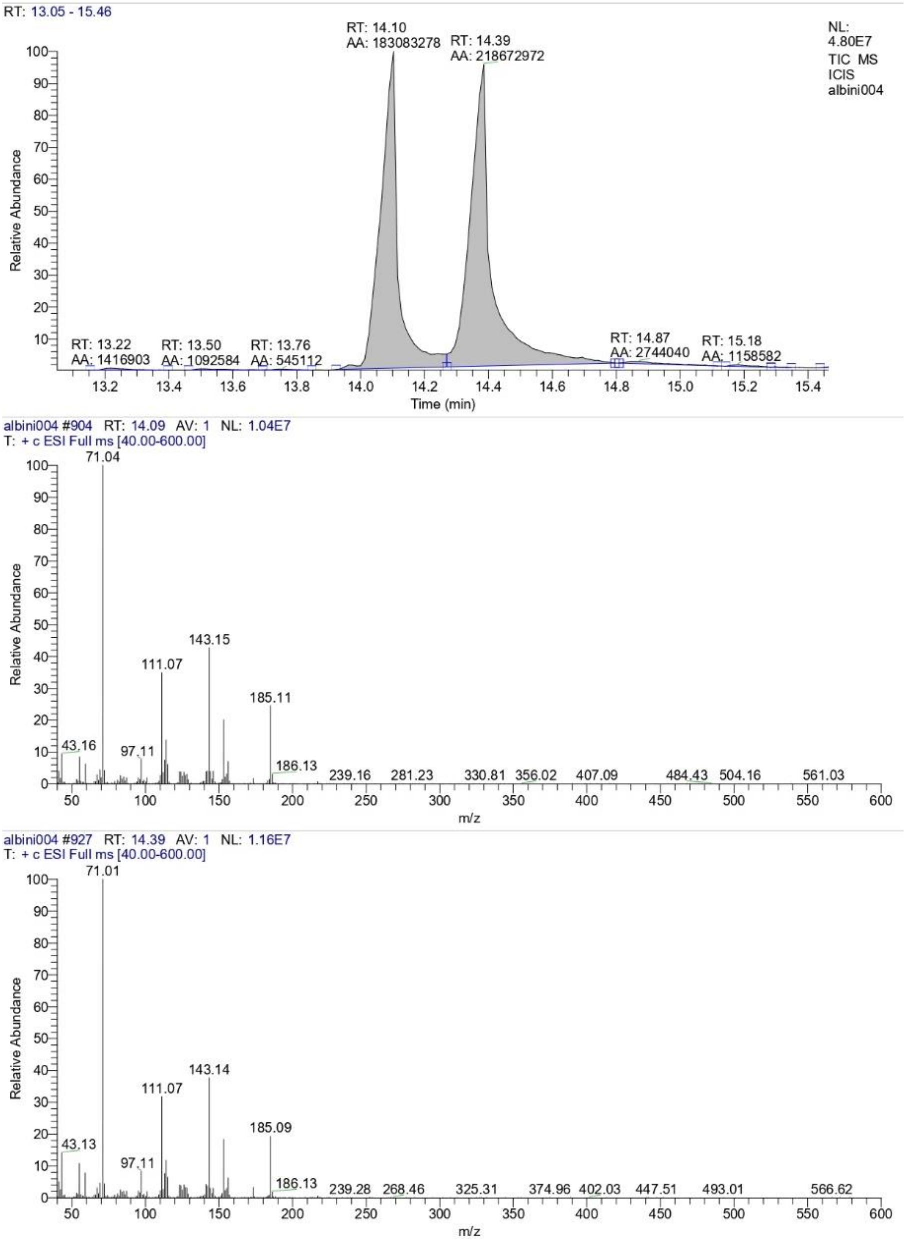


**Figure S7**. Experiment run in the presence of deuterated acetonitrile. Upper part: scheme of the experiment; lower part: particular of the chromatogram of the reaction crude. GC-MS analysis shows that only **3** is formed.

## Experiment in deuterated water

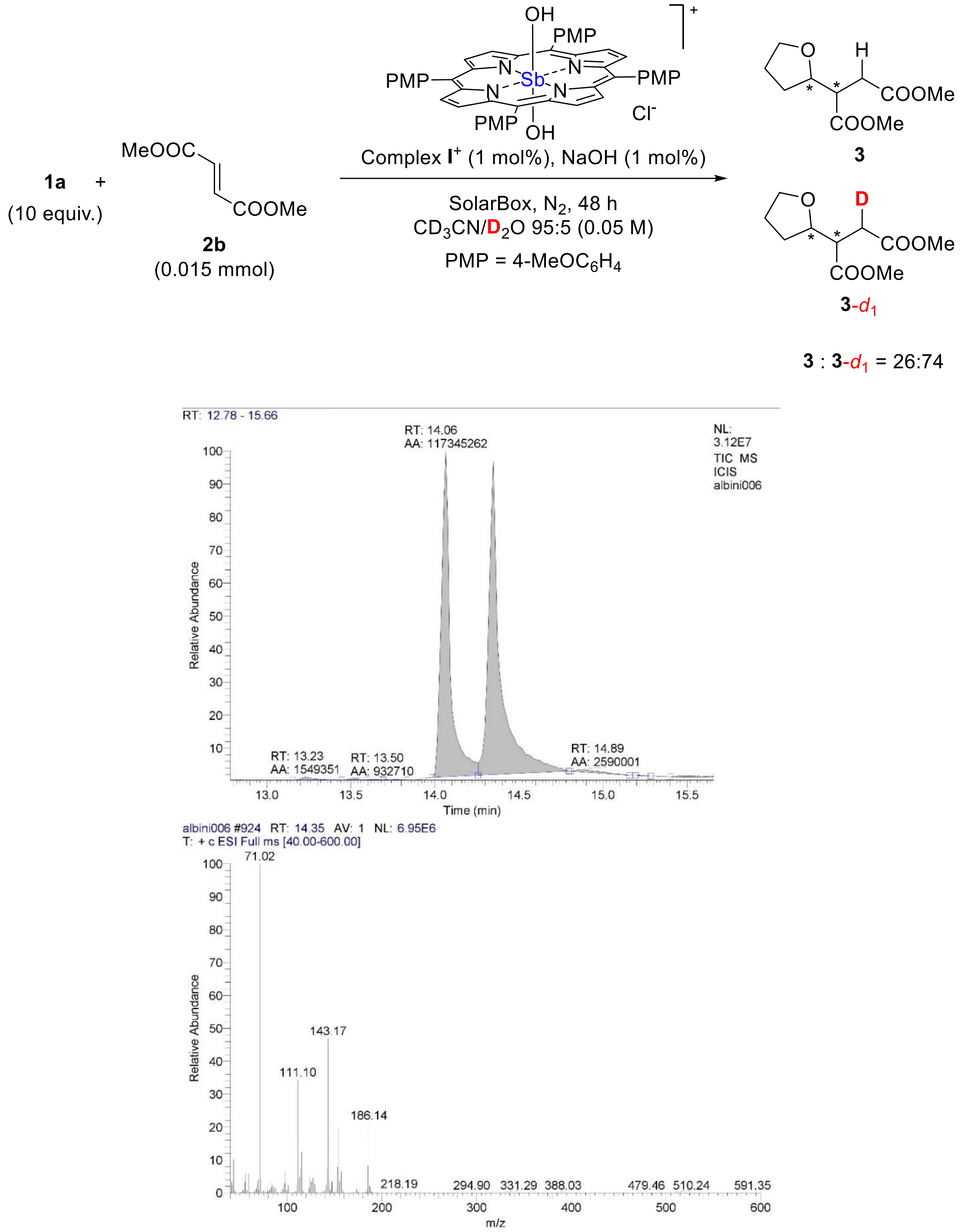


**Figure S8**. Experiment run in the presence of deuterated water. Upper part: scheme of the experiment; lower part: GC-MS analysis showing that **3**-$d_1$ is preferentially formed. The amounts of **3** and **3**-$d_1$ have been determined by comparison of the isotopic distribution of the mass [(M-OMe)$^+$] peaks at *m/z* 185-186.

## 2.4 Photochemical quenching experiments

**Fluorescence quenching tests of the lowest-lying singlet excited state of the complex *oxo*-I:**

Luminescence studies of the antimony-oxo porphyrin samples were carried out in solution in sealed 1-cm cells at room temperature using a Horiba Jobin Yvon Fluorolog-3 spectrofluorometer equipped with two double grating monochromators, a R928P photomultiplier, and an FL-1040 phosphorimeter. All emission spectra measured were corrected for wavelength-dependent instrument and detector response and verified by collecting the corresponding excitation spectra. Relative emission quantum yields were obtained by comparing the areas of the integrated luminescence signals after correction for detector counts of the baseline in the registered spectral region.

No indication for a quenching process of the $S_1$ ($\pi$-$\pi^*$) excited state of *oxo*-**I** by the added hydrogen atom donor THF in concentrations up to 1 M was found in these experiments, which is in contrast to the rich and efficient reductive quenching of the lowest-lying singlet excited state of high-valent antimony porphyrin complexes occurring via a photoinduced electron transfer mechanism.[S7]

**Quenching of the lowest-lying triplet excited state of the complex *oxo*-I:**

Nanosecond laser flash photolysis studies were conducted on an LKS-80 kinetic spectrometer (Applied Photophysics) equipped with a Nd:YAG laser (Quantel) and accessories for transient spectroscopy. Solutions were degassed by repeated freeze-pump-thaw cycles and measured in gas-tight quartz cells with 1-cm path length at ambient temperature. Excitation of the samples was carried out at $\lambda_{exc}$ = 532 nm obtained by second harmonic generation with 110-120 mJ per shot and a laser pulse length of 4-6 ns. Quenching of the lowest-lying triplet state manifold of the complex *oxo*-**I** was monitored by following the decay kinetics of the transient absorption spectrum in the 280-520 nm spectral region.

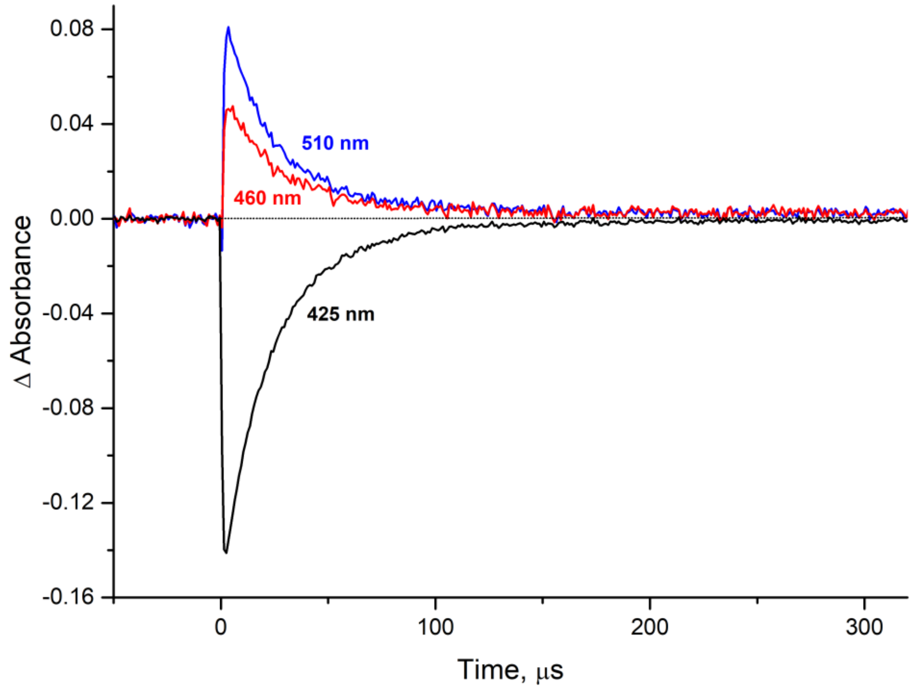


**Figure S9**. Time absorption profiles of *oxo*-**I** in degassed $CH_3CN/H_2O$ 95:5 recorded over a longer time period with respect to Figure 4 (see main text) to visualize the decay of the excited triplet state manifold (transient absorption bands at 460 nm and 510 nm) and the recovery of the corresponding ground state spectrum of *oxo*-**I** as monitored by the full recovery of the bleached Soret band signal at 425 nm.

# 3) References


S1 Gaussian 16, Revision B.01, Frisch, M. J.; Trucks, G. W.; Schlegel, H. B.; Scuseria, G. E.; Robb, M. A.; Cheeseman, J. R.; Scalmani, G.; Barone, V.; Petersson, G. A.; Nakatsuji, H.; Li, X.; Caricato, M.; Marenich, A. V.; Bloino, J.; Janesko, B. G.; Gomperts, R.; Mennucci, B.; Hratchian, H. P.; Ortiz, J. V.; Izmaylov, A. F.; Sonnenberg, J. L.; Williams-Young, D.; Ding, F.; Lipparini, F.; Egidi, F.; Goings, J.; Peng, B.; Petrone, A.; Henderson, T.; Ranasinghe, D.; Zakrzewski, V. G.; Gao, J.; Rega, N.; Zheng, G.; Liang, W.; Hada, M.; Ehara, M.; Toyota, K.; Fukuda, R.; Hasegawa, J.; Ishida, M.; Nakajima, T.; Honda, Y.; Kitao, O.; Nakai, H.; Vreven, T.; Throssell, K.; Montgomery, J. A., Jr.; Peralta, J. E.; Ogliaro, F.; Bearpark, M. J.; Heyd, J. J.; Brothers, E. N.; Kudin, K. N.; Staroverov, V. N.; Keith, T. A.; Kobayashi, R.; Normand, J.; Raghavachari, K.; Rendell, A. P.; Burant, J. C.; Iyengar, S. S.; Tomasi, J.; Cossi, M.; Millam, J. M.; Klene, M.; Adamo, C.; Cammi, R.; Ochterski, J. W.; Martin, R. L.; Morokuma, K.; Farkas, O.; Foresman, J. B.; Fox, D. J. Gaussian, Inc., Wallingford CT, 2016.

S2 Yu, Y.-Q; Wang, Z.-L. A simple, efficient and green procedure for Knoevenagel condensation in water or under solvent-free conditions. *J. Chin. Chem. Soc.* **2013**, *60*, 288–292.

S3 Ertl, M.; Wöβ, E.; Knör, G. Antimony porphyrins as red-light powered photocatalysts for solar fuel production from halide solutions in the presence of air. *Photochem. Photobiol. Sci.* **2015**, *14*, 1826–1830.

S4 Rohe, S.; Morris, A. O.; McCallum, T.; Barriault, L. Hydrogen Atom Transfer Reactions via Photoredox Catalyzed Chlorine Atom Generation. *Angew. Chem. Int. Ed.* **2018**, *57*, 15664–15669.

S5 Bonassi, F.; Ravelli, D.; Protti, S.; Fagnoni, M. Decatungstate Photocatalyzed Acylations and Alkylations in Flow via Hydrogen Atom Transfer. *Adv. Synth. Catal.* **2015**, *357*, 3687–3695.

S6 Capaldo, L.; Merli, D.; Fagnoni, M.; Ravelli, D. Visible light uranyl photocatalysis: direct C–H to C–C bond conversion. *ACS Catal.* **2019**, *9*, 3054–3058.

S7 Knör, G. Reductive fluorescence quenching of the photoexcited dihydroxy antimony(V) tetraphenylporphine cation in acetonitrile solution. *Chem. Phys. Lett.* **2000**, *330*, 383–388.
